# Multisite Threonine Phosphorylation in the SPT5 C‑Terminal Region 1 Enables Sequence-Dependent Regulation without Global Structural Remodeling

**DOI:** 10.1021/acs.jpcb.6c03886

**Published:** 2026-08-10

**Authors:** Rong Hu, Emery T. Usher, Olivia A. Fraser, Wei Chen, Alex S. Holehouse, Scott A. Showalter

**Affiliations:** † Department of Chemistry, 8082 The Pennsylvania State University, University Park, Pennsylvania 16802, United States; ‡ Department of Chemistry and Biochemistry, 21769 University of California, Santa Cruz, California 95064, United States; § Center for Eukaryotic Gene Regulation, Department of Biochemistry and Molecular Biology, The Pennsylvania State University, University Park, Pennsylvania 16802, United States; ∥ Department of Biochemistry and Molecular Biophysics, Washington University School of Medicine, St. Louis, Missouri 63110, United States; ⊥ Center for Biomolecular Condensates, 7548 Washington University in St. Louis, St. Louis, Missouri 63110, United States

## Abstract

Phosphorylation of intrinsically disordered regions (IDRs) is widely viewed as a mechanism for encoding regulatory information through structural transitions, as exemplified by the RNA polymerase II C-terminal domain (CTD), where phosphorylation induces local conformational changes such as proline isomerization. However, whether this paradigm extends to other disordered regulatory domains remains unclear. The C-terminal repeat region (CTR) of SPT5, a key cofactor in transcription elongation, contains multiple Thr–Pro motifs that are phosphorylated by P-TEFb, but the structural and mechanistic consequences of this modification have not been defined. Here, we combine NMR spectroscopy, small-angle X-ray scattering (SAXS), mass spectrometry, and molecular simulations to characterize the effects of multisite threonine phosphorylation on the SPT5 CTR1 domain. NMR analysis reveals that, in contrast to the RNA polymerase II CTD, phosphorylation does not induce detectable proline isomerization. Molecular simulations suggest this resistance arises because phosphothreonine forms an intramolecular hydrogen bond that restricts the backbone flexibility required for isomerization. SAXS measurements further show that hyperphosphorylation does not alter the global dimensions of the CTR1 ensemble, in agreement with predictions from polyampholyte polymer models. Notably, mass spectrometry and mutational analysis demonstrate that phosphorylation is strongly influenced by local sequence context, with specific residues modulating kinase selectivity. Together, these findings establish that multisite phosphorylation of the SPT5 CTR1 domain modulates a heterogeneous interaction landscape shaped by sequence context. More broadly, this work emphasizes the nontrivial difference between serine and threonine phosphorylation as a mechanism to regulate disordered protein structure–function relationships.

## Graphical abstract

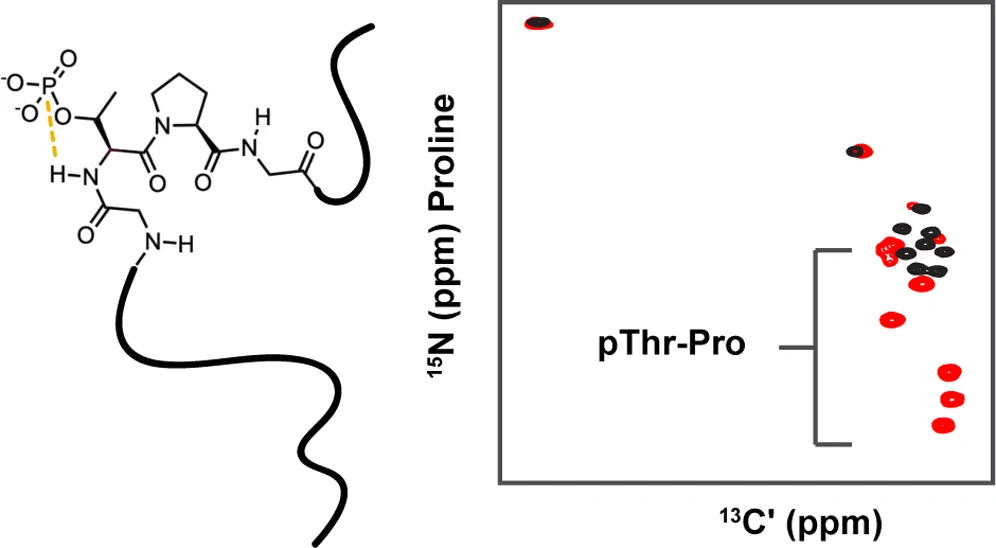

## Introduction

During most transcription events, RNA polymerase II (Pol II) pauses tens of nucleotides downstream of the transcription start site, a process mediated by DRB sensitivity-inducing factor (DSIF) and negative elongation factor (NELF).^1–4^ Phosphorylation by the positive transcription elongation factor b (P-TEFb), a cyclin-dependent kinase complex, converts DSIF into a positive elongation factor and triggers NELF dissociation, allowing productive elongation to proceed.^3,5–10^ These regulatory transitions are primarily coordinated through phosphorylation of Thr–Pro pairs in the SPT5 C-terminal region (CTR), suggesting that intrinsically disordered regions act as key platforms for dynamic transcriptional control.^11–14^

Outside of the transcription community, the RNA Pol II C-terminal domain (CTD) serves as a better known model for how phosphorylation of disordered repeat sequences can encode regulatory information.^5,15,16^ Changes in CTD phosphorylation patterns correlate with transcriptional stage transitions and cofactor recruitment.^17,18^ In some sequence contexts, CTD phosphorylation also induces local proline isomerization, highlighting how structure–function relationships can be maintained in the face of overall protein disorder.^19,20^ These insights raise the possibility that other intrinsically disordered regions with repetitive primary structures, such as the SPT5 CTR, may follow similar principles.

The SPT5 CTR is an intrinsically disordered region (IDR) that is conceptually subdivided into two domains named CTR1 and CTR2, respectively, which differ in both sequence composition and regulatory function.^21–23^ Although these domains form a continuous IDR, functional studies have shown they exert opposing effects on transcription: threonine phosphorylation in CTR1 promotes pause release and accelerates productive elongation, whereas the presence of CTR2 restrains elongation by limiting CTR1 phosphorylation or directly slowing elongation kinetics.^22,23^ CTR1 contains nine pentapeptide repeats with variable levels of conformity to the consensus sequence G-S-R/Q-T-P, dispersed between spacers with lower sequence conservation.^5^ Similar repeat architectures are found in SPT5 across metazoans, where this region serves as a major target of the kinase P-TEFb.^5,21,24,25^ In contrast, CTR2 is composed of ten more variable repeats with a distinct consensus motif (P–T/S–P–S–P–Q/A–S/G–Y), similarly dispersed between spacer sequences of varying length, and is generally considered a poor substrate for P-TEFb.^21–23^ These characteristics motivate a residue-level study of SPT5 CTR1 to establish regulatory structure–function relationships driven by threonine hyper-phosphorylation in proteins.

Here we performed a comprehensive biophysical analysis of the *Drosophila melanogaster* CTR1 domain. We chose to study the *Drosophila* SPT5 CTR because greater sequence variation within the repeats, relative to the human sequence, facilitates unambiguous study through nuclear magnetic resonance (NMR), while the overall domain architecture and sequence characteristics are generally well conserved.^26^ Using NMR spectroscopy, we identified six specific Thr–Pro sites within CTR1 that are well phosphorylated by P-TEFb. We find differences in local proline isomerization, relative to those induced by serine phosphorylation of Pol II CTD, which suggest significant mechanistic differences between threonine and serine phosphorylation. Small angle X-ray scattering (SAXS) analysis further revealed that hyper-phosphorylation does not cause substantial global conformational changes, which is like the outcome for CTD,^19,20^ despite the differences in their overall charge and aromatic residue distribution. Insight into these results is gained from ensemble modeling, which suggests that insensitivity of the CTR1 ensemble to global changes induced by phosphorylation arises largely from the positive residues in the sequence. Finally, mass spectrometry profiling suggests that local sequence context, including adjacent tyrosine residues, may bias phosphorylation at certain sites. Together, our findings shed light on how intrinsic sequence variation within disordered repeats, specifically those in which threonine hyperphosphorylation is a feature, shape regulatory behavior in transcriptional elongation.

## Methods

### Plasmids

*Escherichia coli* codon-optimized DNA sequence encoding residues 796 to 972 (full-length CTR) of the *D. melanogaster* SPT5 (Uniprot Q9 V460) was inserted into the pET His6MBP TEV LIC cloning vector (1M, Addgene plasmid #29656) using ligation independent cloning. The plasmid sequence was verified at the Penn State Genomics Core Facility.

*E. coli* codon-optimized DNA sequence encoding residues 796 to 881 (CTR1) of the *D. melanogaster* SPT5 was inserted into the pET His6MBP TEV LIC cloning vector (1M, Addgene plasmid #29656) using ligation independent cloning. All mutagenesis was accomplished with Q5 site directed mutagenesis (NEB E0554S), with primers designed via NEBaseChanger and procured from Integrated DNA Technologies. Mutants generated included Y811A, Y843A, Y811/Y843A, V828Y, R803Q, R807Q, R840Q, R803/807/840Q, G812D, D838G, D838A, and D838L. All plasmid sequences were verified at the Penn State Genomics Core Facility.

### SPT5 Protein Expression in *E. coli*

BL21 (DE3) cells containing the plasmid required for each protein were grown at 37 °C in either M9 minimal media for NMR experiments, or Luria broth (LB) media for all other experiments, in all cases supplemented with 50 μg/mL kanamycin. When generating NMR samples, the M9 media was prepared with ^13^C and ^15^N enrichment achieved through the incorporation of ^13^C d-glucose and ^15^N-ammonium chloride (Cambridge Isotope Laboratories). At an optical density at 600 nm of 0.8 to 1.0, expression was induced with 0.5 mM isopropyl β-1-thiogalactopyranoside (IPTG) at 37 °C for 3 h. Following purification, samples were buffer exchanged and concentrated in an Amicon Ultra Centrifugal Filter with a 3 kDa molecular weight cutoff (MilliporeSigma), yielding a final concentration of ∼0.8 mM in 50 mM imidazole, 200 mM KCl, and 10% D_2_O. Cells were pelleted by centrifugation at 4200*g* for 30 min and washed with 20 mM *Tris* pH 7.5, 20 mM NaCl, and 2 mM EDTA.

### Protein Purification

Cell pellets were resuspended in the lysis buffer (50 mM *Tris* pH 7.5, 500 mM NaCl, 20 mM imidazole, and 2.5 mM β-mercaptoethanol) supplemented with 1× ethylenediaminetetraacetic acid (EDTA)-free protease inhibitor cocktail (Millipore) and 1 mM phenylmethylsulfonyl fluoride (PMSF). Following sonication on ice, the cell lysate was centrifuged at 4 °C for 45 min at 16,500*g*. The supernatant was passed through Ni-NTA resin (Thermo Fisher Scientific) equilibrated with the lysis buffer and washed with 5 column volumes of the lysis buffer. Proteins were eluted in 2 column volumes of elution buffer (50 mM *Tris* pH 7.5, 500 mM NaCl, 200 mM imidazole, and 2.5 mM β-mercaptoethanol). The eluate was incubated with TEV protease (2 mg per 1 L growth) under dialysis against 2 L of 50 mM *Tris* pH 7.5, 100 mM NaCl, 20 mM imidazole, and 2.5 mM β-mercaptoethanol at 4 °C for 16 h. TEV cleavage leaves a short N-terminal artifact (SNA) in the final protein sequence. Given the intrinsically disordered nature of the constructs used in this study and the absence of structured elements at the N-terminus, this additional sequence is not expected to significantly influence the conformational ensemble or phosphorylation behavior. Dialysates were passed through a second Ni-NTA column, and the flow-through containing target protein was collected and concentrated using an Amicon Ultra Centrifugal Filter with a 3 kDa molecular weight cutoff (MilliporeSigma).

### P-TEFb Expression in Sf9 Cells

The *Drosophila* P-TEFb complex is minimally reconstituted in vitro from cyclin dependent kinase-9 (Uniprot O17432) and cyclin T (Uniprot O96433). Sf9 cells were grown in suspension at 27 °C to 2 million cells mL^–1^ and infected with 1/10 culture volume *D. melanogaster* P-TEFb virus (generous gift from J.T. Lis). Infection was carried out at 27 °C at a shaker speed of 125 rpm for 72 h. Cells were pelleted by centrifugation at 200*g* for 10 min.

### P-TEFb Purification

Cell pellets were resuspended in the lysis buffer (50 mM HEPES pH 7.5, 500 mM NaCl, 2.5 mM imidazole, 10% glycerol, 1% Nonidet P-40, and 2.5 mM β-mercaptoethanol), supplemented with 1 x EDTA-free protease inhibitor cocktail (Millipore). Following lysis by dounce homogenization. The cell lysate was centrifuged at 4 °C for an hour at 100,000*g*. Cleared supernatant was mixed with TALON resin (Clontech) equilibrated with lysis buffer and incubated with agitation at 4 °C for an hour. Bound protein was washed using 5 column volumes of lysis buffer and eluted with 50 mM HEPES pH 7.5, 500 mM NaCl, 10% glycerol, 1% Nonidet P-40, 200 mM imidazole and 2.5 mM β-mercaptoethanol and stored at −80 °C. Kinase activity toward CTR constructs was confirmed by MALDI-TOF mass spectrometry.

### Protein Phosphorylation

Phosphorylation reactions were carried out in 50 mM imidazole, 100 mM KCl, 20 mM MgCl_2_, 2 mM DTT, 10 mM ATP with 0.2 mg/mL Dm P-TEFb and 250 μM proteins. Reactions were incubated at 25 °C for 48 h to proceed to completion. For MALDI-TOF analysis, reactions were quenched with 1% trifluoroacetic acid. For NMR analysis, reactions were quenched with 20 mM EDTA.

### MALDI Data Collection

Samples of intact unphosphorylated and phosphorylated CTR1 constructs were desalted using G-Biosciences C-18 Spin Columns and desiccated in a SpeedVac Vacuum Concentrator. Mass spectra were acquired on a Bruker UntrafleXtreme MALDI TOF/TOF instrument using the factory linear positive-ion method. The *m/*z detection range was 5000 to 20,000. Dried samples were resuspended in 5 μL of water prior to analysis.

The matrix consisted of 20 mg/mL super-DHB (Sigma p/n 50,862-1G-F) in 50% acetonitrile, 0.1% trifluoroacetic acid, and 0.1% phosphoric acid, and was mixed with sample at a 1:1 volume ratio. Approximately 1 μL of sample-matrix mixture was applied to a polished steel target. Laser power attenuation and pulsed ion extraction time were optimized for signal quality.

### MALDI Data Analysis

Spectra were smoothed (Savitzky–Golay, width 5 *m*/*z*, one cycle) and baseline-subtracted using the TopHat algorithm. Mass lists were generated using a centroid peak-detection algorithm. Processed spectra were exported and plotted in R for visualization and comparison. Spectra are shown over the mass range corresponding to intact CTR1 and its phosphorylated species, and low-intensity fragment peaks and background signals were excluded from display for clarity.

### Simulations

All-atom simulations of small pSer- and pThr-containing peptides (“Ser”—G–S–P–G; “Thr”—G–T–P–G; “pSer”—G-pS–P–G; “pThr”—G–pT–P–G) were performed using the CAMPARI simulation engine and ABSINTH/OPLS-AA implicit solvent model.^27,28^ The peptide sequences were capped at the N- and C-termini with acetyl and amide groups, respectively, and each simulated inside a spherical droplet with radius = 25 Å at 300 K. The monovalent ion concentration (NaCl) was set to 0.1 M and was modeled explicitly. Each independent simulation consisted of 50 million steps with write-out every 20,000 frames; the first 2.5 million frames of each trajectory were discarded as equilibration. With five replicates per sequence, each merged trajectory ultimately contained 11,875 frames.

Conformational ensembles of CTR1 and variants were predicted from amino acid sequence (Table S1) using the tool STARLING.^29^ Each ensemble consists of 2000 independent protein conformations, and Glu (E) was used in place of pThr at the necessary sites to mimic phosphorylation. Net charge per residue (NCPR) and fraction of charged residues (FCR) profiles were calculated for the CTR1 and eCTR1 (phosphomimetic) sequences using the tool CIDER.^30^ Average NCPR and FCR, as well as the charge patterning parameter, kappa, are given for each STARLING-predicted ensemble in Table S1.

### Simulation Data Analysis

Peptide simulations were analyzed in Python using the MDTraj package to measure interatomic distances and root-mean-square deviation (RMSD) of functional groups.^31^ In particular, the structures were aligned then the mean of the positions of the six atoms of the phosphate moiety (OG1, P, OH, O1P, O2P, HOP, where the atom type is underlined) were tracked over all simulation frames with reference frame 1. Hydrogen bond occupancy was determined using “strict” and “relaxed” critera. The “strict” condition requires that atoms be within 2.5 Å and have a bond angle of >150°; the ‘relaxed’ condition requires that atoms be within 2.5 Å and have a bond angle of >120°. Occupancy was compared between pSer and pThr for both strict and relaxed cases using a Fisher’s exact test. Given the number of data points, exact *p* values fell below floating-point precision and so are described simply as *p* < 0.001. STARLING ensembles were analyzed using the MDTraj and SOURSOP packages.^31,32^ SAXS profiles were calculated from the STARLING ensembles using FoXS to yield 200 profiles each.^33^ The experimental and calculated scattering curves were scaled and their quantitative agreement in the q range of 0.009 to 0.350 was determined using the parameter chi-squared free (Χ^2^_free_).^34^

### NMR Data Collection

NMR data were collected in the Lloyd Jackman Nuclear Magnetic Resonance Facility at the Pennsylvania State University on Bruker Avance NEO 600 MHz spectrometer equipped with a triple resonance TCI singe-axis gradient cryoprobe. All NMR data were acquired at 25 °C using TopSpin (Bruker). A total of 1024 complex points were collected in the direct dimension for all experiments. The experimental design used ^13^C direct-detect methods as previously described,^35,36^ supplemented by proline-selective spectra noted below. For two-dimensional experiments, 256 complex points were collected in the indirect dimension; for three-dimensional experiments, 64 × 128 complex points were collected in the indirect dimensions. Unless otherwise noted, sweep widths were 54 ppm for ^15^N, 30 ppm for backbone ^13^C, and 80 ppm for side-chain ^13^C, with carrier frequencies centered at 173 ppm (^13^C) and 124 ppm (^15^N). CON and (HACA)­N­(CA)­NCO experiments were acquired with 16 scans, while (HACA)­N­(CA)­CON and (H)­CCCON were acquired with 8 scans. Proline-selective (HACA)­CON was acquired with 8 scans and reduced sweep widths of 19 ppm (^13^C) and 12 ppm (^15^N).^37^ Proline-selective (H)­CCCdNCO and (H)­CCCON were acquired with 8 scans and reduced ^15^N sweep widths of 10 and 12 ppm, respectively.^38^ All proline-selective experiments used carrier frequencies centered at 170 ppm (^13^C) and 139 ppm (^15^N).

### NMR Data Analysis

Data were processed using TopSpin (Bruker). Chemical shift assignments were carried out manually in POKY^39^ using our standard protocol.^40^ Chemical shifts were referenced indirectly to DSS according to IUPAC recommendations.^41^

### SAXS Data Collection

Native CTR1 was purified as described above in a final buffer of 50 mM *Tris* pH 7.5, 100 mM NaCl, and 20 mM imidazole. Phosphorylated CTR1 was prepared as described above, followed by heat treatment at 65 °C for 5 min to remove P-TEFb, and subsequently buffer exchanged into the same buffer as the native sample. SAXS measurements were performed at protein concentrations of 2 and 4 mg/mL.

SAXS data were collected using a Rigaku MM007 rotating anode X-ray source coupled to a BioSAXS2000nano Kratky camera system equipped with OptiSAXS confocal max-flux optics and a HyPix-3000 Hybrid Photon Counting detector. The sample-to-detector distance was calibrated to 495.5 mm using silver behenate.^42^ Data were collected over a momentum transfer range of *q* = 0.009–0.6 Å^–1^ (*q* = 4πsin (θ)/λ, where 2θ is the scattering angle).^43^ The X-ray beam energy was set to 1.2 keV, with a beam diameter of approximately 100 μm and attenuation of 22%.

Protein samples were loaded into a quartz capillary flow cell using the Rigaku autosampler. The beam path and detector chamber were maintained under vacuum (<1 × 10^–3^ Torr) to minimize air scattering. Data acquisition and automated cleaning cycle were controlled using SAXSLAB software (Rigaku).

### SAXS Data Analysis

Data processing was performed using SAXSLAB software (Rigaku). For each condition, scattering profiles were averaged from 6 to 10 min exposures collected across two independent replicates of both protein and buffer samples. Overlays of sequential exposures confirmed the absence of radiation damage. Buffer subtraction was performed to obtain the protein scattering profile.

Further analysis was carried out using the ATSAS 4 package.^44^ Pair-distance distribution functions P­(r) were calculated using GNOM following established protocols for intrinsically disordered proteins, with *D*_max_ selected such that P­(r) smoothly approaches zero at large r.^45,46^ Processed data were visualized and plotted in R.

## Results and Discussion

### Comparison between Full-Length CTR and CTR1

Before examining phosphorylation and structural properties of the isolated CTR1 construct (86 residues), we first assessed whether truncation of the C-terminal repeat region alters its local chemical environment relative to the same residues within full-length SPT5 CTR (177 residues). A schematic representation of the SPT5 domain architecture and the location of the CTR1 construct within the full-length CTR is shown in Figure 1A. Overlay of the ^13^C,^15^N–CON spectra of isolated CTR1 (black) with that of full-length CTR (blue) reveals highly similar resonance positions across the two constructs, including both nonproline residues and proline-selective correlations (Figure 1B,C), indicating that the local backbone environments are largely preserved upon truncation. Quantitative analysis of chemical shift perturbations confirms that differences between the constructs are small and randomly distributed along the sequence, except for residues near the newly generated C-terminus of CTR1 (Figure S1). These observations indicate that the conformational ensemble of CTR1 closely resembles that of the corresponding region within the full-length CTR, supporting the use of CTR1 as a representative construct for the structural and phosphorylation analyses described below.

**Figure 1:**
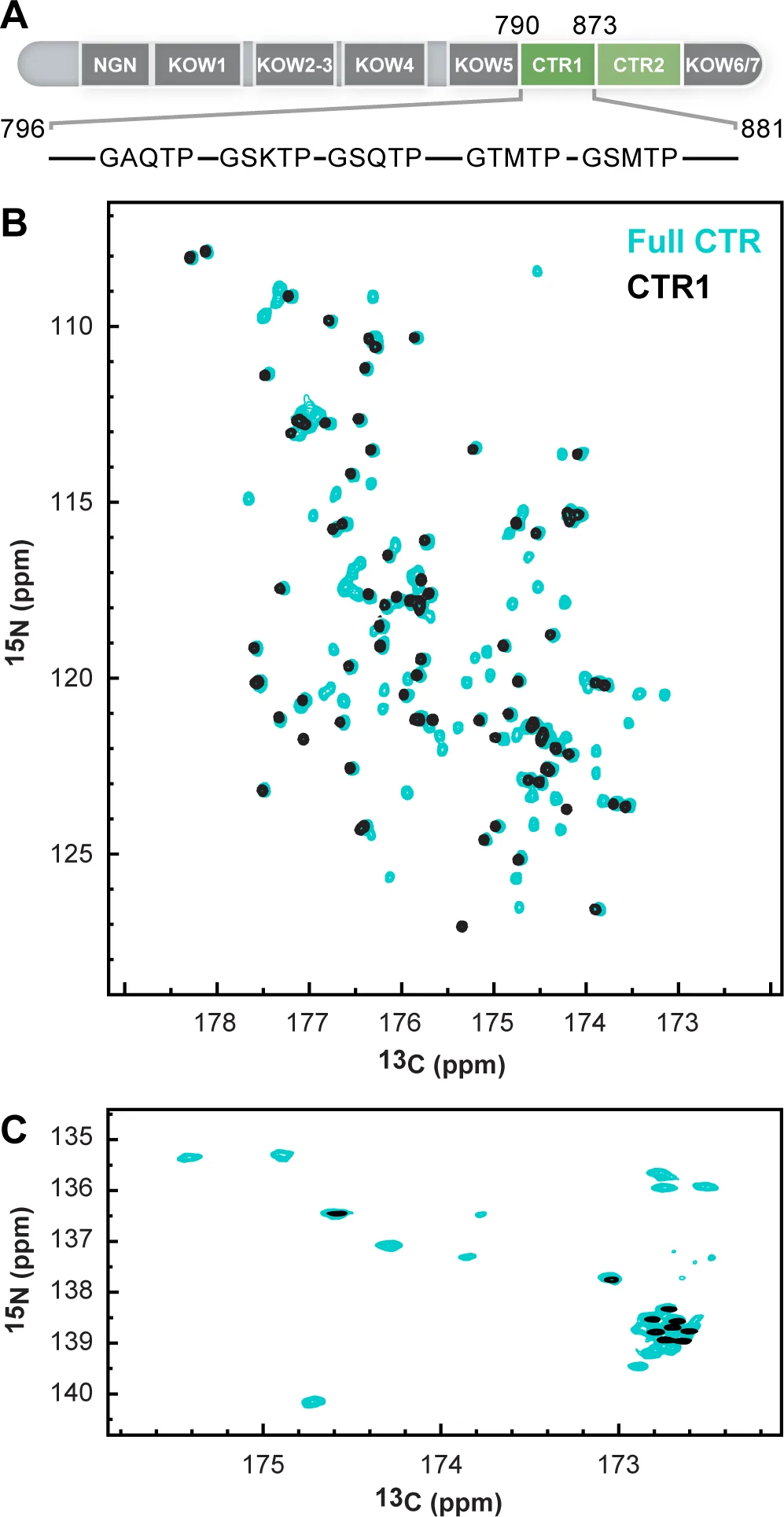
NMR demonstrates a highly similar chemical environment for SPT5-CTR1 and the same residues in the context of full-length SPT5-CTR. (A) Schematic of the SPT5 domain structure, highlighting five motifs in the CTR1 region that correspond well to the consensus Thr–Pro containing pentapeptide. Overlay of (B) the ^13^C,^15^N–CON spectra, cropped to display nonproline residues, and (C) the proline-selective ^13^C,^15^N–CON spectra for SPT5-CTR1 (black) and full-length SPT5-CTR (blue).

### P-TEFb Phosphorylates Multiple Threonine Sites in CTR1

Here we set out to establish the phosphorylation profile of the native SPT5-CTR1 region and subsequently evaluate the influence of threonine phosphorylation on its solution conformational ensemble. CTR1 encodes nine G–S–R/Q–T–P motifs, each containing a Thr4-Pro5 pair that represents a potential kinase substrate but otherwise deviating from the consensus to varying degrees, presented in the context of flanking sequences to facilitate comparison with the RNA Pol II CTD heptamer (Figure 2A). To begin, we established the extent and heterogeneity of CTR1 phosphorylation by P-TEFb using MALDI-TOF mass spectrometry. Mass spectra from the unphosphorylated and phosphorylated samples revealed a distribution of species bearing 5–9 phosphates, with the +7 state most abundant (Figure 2B). We observed the same distribution across independent preparations of both CTR1 and P-TEFb, confirming that the observed heterogeneity reflects reproducible selectivity in the phosphorylation reactions rather than experimental variation. While MALDI-TOF is not formally a quantitative technique, one of its advantages is sensitivity to low-population species present in the measured sample. Therefore, this qualitative pattern is consistent with extensive multisite modification but highlights the persistence of some sample heterogeneity that will need to be accounted for in analysis of the remaining studies reported here. Similar incomplete or sequence-biased phosphorylation has been reported for other disordered repeat domains, including the *Drosophila* Pol II CTD, which remains partially modified even under extensive kinase treatment,^19,20,47,48^ and other IDRs such as Ash1 and FUS LC.^49,50^ Even after prolonged incubation with excess kinase and ATP/Mg^2+^, the reaction did not reach full product homogeneity, indicating that several Thr–Pro sites are intrinsically poor substrates for P-TEFb and imply a level of sequence selectivity within CTR1.

**Figure 2:**
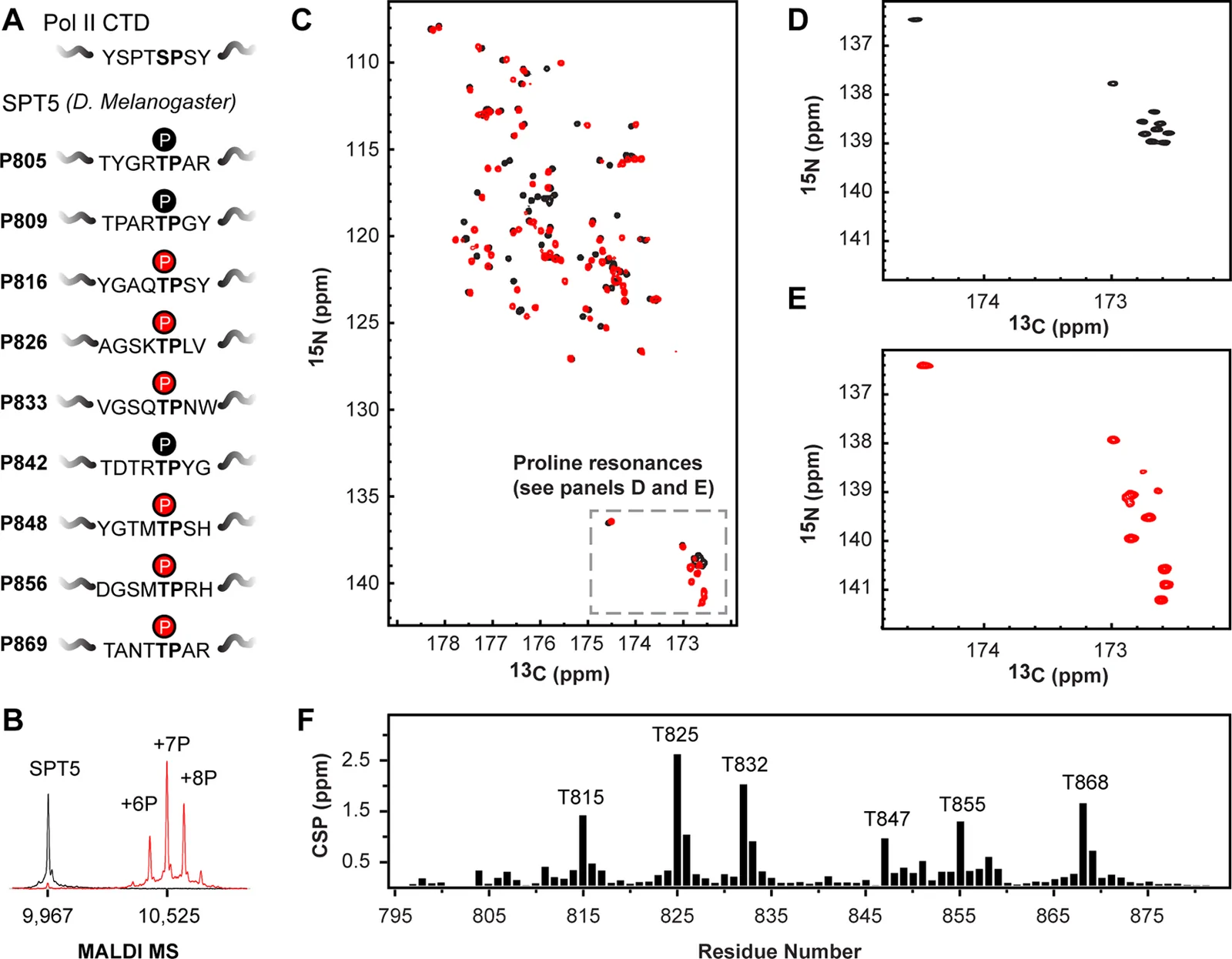
Identification of six Thr–Pro motifs in the SPT5-CTR1 region that undergo stoichiometric phosphorylation by P-TEFb in solution. (A) SPT5 encodes Thr–Pro in pentapeptide repeats that bear similarity to the RNA Pol II CTD heptad repeat. While nine repeats are found in *D. melanogaster* CTR (residues 796–881), our NMR data indicate stoichiometric phosphorylation of six (red circle). (B) MALDI-TOF MS reveals 6–8 phosphorylation events, consistent with both the complete phosphorylation at six sites and partial resonance intensity reduction in two of the other three motifs. (C) ^13^C, ^15^N–CON spectrum of SPT5 CTR1 (black) overlaid by the same spectrum of phosphorylated SPT5 CTR1 (red). (D) Proline-selective ^13^C, ^15^N–CON spectrum, revealing near baseline resolution of all ten resonances. (E) Following P-TEFb treatment, pro-selective CON displays large chemical shift changes for six resonances, while two others feature reduced resonance intensity, suggestive of partial phosphorylation. (F) ^13^C, ^15^N–CON chemical shift perturbations indicate the six threonine residues that undergo stoichiometric phosphorylation.

We next sought to determine which threonine residues within the CTR1 consensus repeats are phosphorylated by P-TEFb. Although P-TEFb is known to recognize Ser/Thr–Pro motifs, the extent to which all repeats are equivalently modified has not been established. It is also noteworthy that CTR1 contains no Ser–Pro pairs; therefore, all the phosphorylation events observed are uniquely associated with Thr–Pro pairs, providing the most extensive set we are aware of in the literature for exploration of this motif.

Site-specific phosphorylation analysis requires the availability of complete NMR chemical shift assignments for both the unphosphorylated and Thr-phosphorylated states of CTR1. Therefore, we employed our standard ^13^C direct-detect NMR spectroscopy approach, augmented with recently optimized proline-selective experiments for superior resolution in this region of the spectrum. Proline-selective experiments were essential for improving spectral dispersion and isolating each Thr–Pro motif in the unphosphorylated sample,^37,38^ because backbone correlations for the proline residues were difficult to distinguish using standard methods (Figure 2C–E). Together, these data sets yielded complete and unambiguous assignments for both states. Fully assigned spectra are provided in the Supporting Information (Figures S2 and S3), and the corresponding assignments have been deposited in Biological Magnetic Resonance Data Bank (BMRB access numbers 53838, unphosphorylated SPT5; 53839, phosphorylated SPT5).

Analysis of the assigned CON spectra revealed pronounced chemical shift perturbations (CSP) in six regions of the CTR1 sequence, each corresponding to a distinct Thr–Pro repeat (Figure 2F). To confirm that these perturbations arose from phosphorylation of the threonine residues, we examined the associated side-chain resonances and observed characteristic downfield shifts of the Thr β-carbon signals, relative to the ^13^Cβ distribution in the BMRB,^51^ consistent with formation of phosphothreonine (Figure 3). Together, these data identify six Thr–Pro motifs that undergo (nearly) complete phosphorylation by P-TEFb, while the remaining repeats exhibit either diminished intensity or minimal perturbation. These observations are consistent with the overall mass heterogeneity observed by mass spectrometry; phosphorylation of the other three Thr–Pro pairs proceeds only to low-population, yielding species that are largely invisible by NMR and a spectrum reflecting the primary phospho-state. In summary, our analysis indicates that P-TEFb does not modify all Thr–Pro pairs from the pentapeptide motifs within CTR1 equivly. Whether the variation originates within the repeats or is driven by surrounding sequence context is explored below.

**Figure 3:**
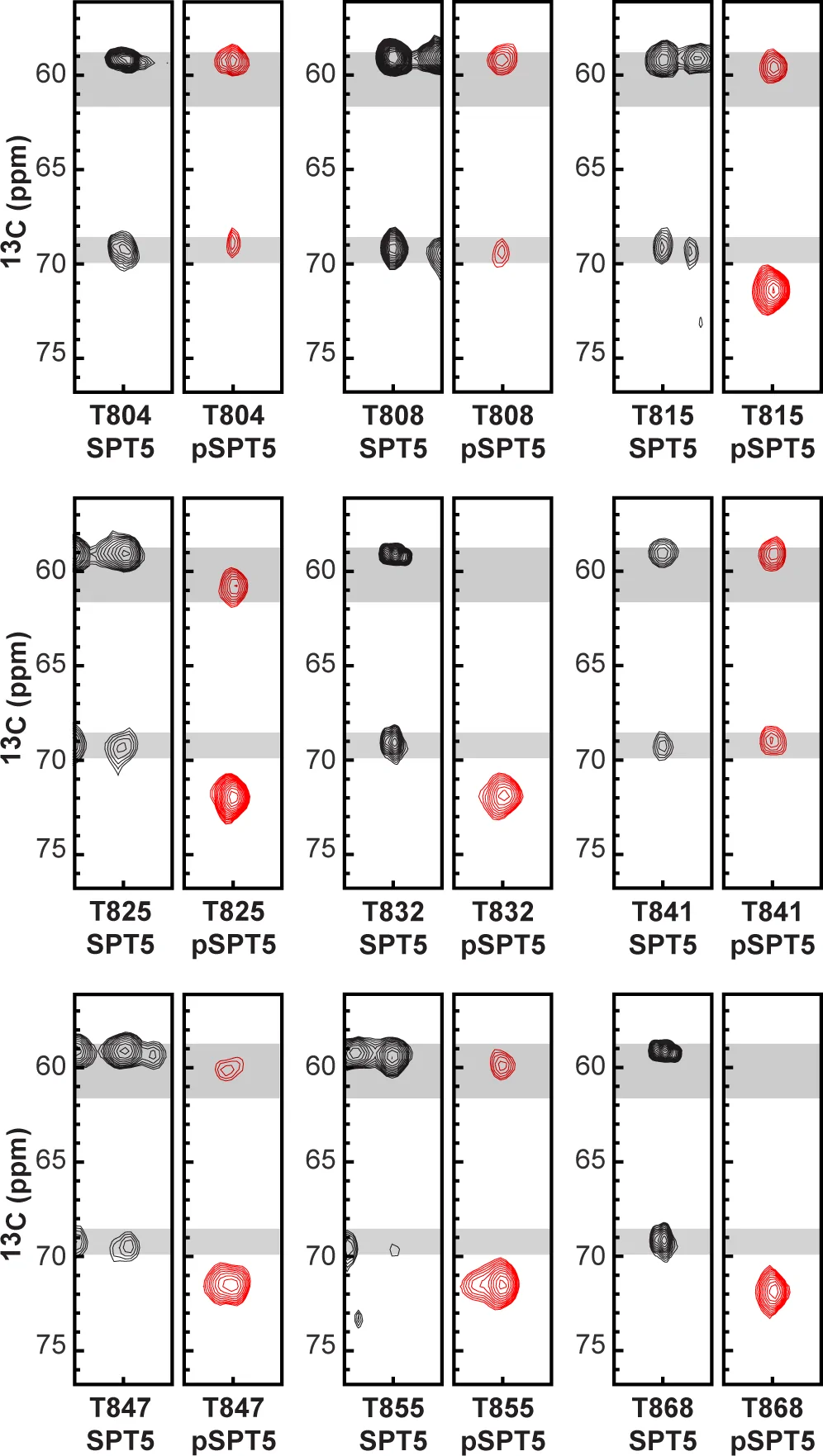
Validation through side chain chemical shift change of the six Thr–Pro motifs in the SPT5-CTR1 region that undergo stoichiometric phosphorylation by by P-TEFb in solution. For each Thr–Pro pair, strip plots from the 3D (H)­CCCON-IPAP spectrum correspond to unphosphorylated (black) and phosphorylated (red) CTR1. Gray bands indicate the expected range of chemical shifts for the threonine ^13^Cα and ^13^Cβ established by the BMRB.^51^ In the phosphorylated sample, ^13^Cβ > 70 ppm is consistent with threonine phosphorylation.

Our findings are consistent with prior studies demonstrating P-TEFb-dependent phosphorylation of the SPT5 CTR, which established Thr–Pro pairs within the repeats as kinase substrates but did not define the extent or distribution of phosphorylation across the repeats.^5^ Previous work identified a limited number of representative sites in the human protein using targeted biochemical assays, while the broader set of CTR repeats has generally been treated as equivalently phosphorylatable.^21,52^ In contrast, our NMR-based strategy enables direct, residue-specific identification of phosphorylation events within an intact CTR1 construct under defined conditions, allowing simultaneous assessment of all repeats within a single molecular context. This approach reveals that P-TEFb selectively phosphorylates a subset of Thr–Pro pairs rather than modifying all repeats equivalently. This provides the first comprehensive, site-resolved mapping of phosphorylation across the *Drosophila* CTR1. Together, these results establish that P-TEFb phosphorylates the majority, but not all, of the potential Thr–Pro pairs in CTR1. This selective modification defines the starting point for evaluating whether phosphorylation has structural consequences for the disordered domain.

### Phosphorylation Does Not Induce Proline Isomerization in CTR1

We next tested whether CTR1 phosphorylation induces proline isomerization, a local structural change previously reported in the Pol II CTD. In the CTD heptad repeat sequence YSPTSPS, phosphorylation of Ser5 shifts the *cis*/*trans* equilibrium of the adjacent Pro6 toward the *cis* state, producing a *cis* population detectable by NMR. This effect is enhanced when Ser7 is substituted for Asn7, as occurs naturally in both *Drosophila* and mammals.^19^ Importantly, this isomerization effect is absent in the one CTD repeat from the data set where Thr replaces Ser at position 5, raising the possibility that phospho-threonine behaves differently. However, it remained unclear whether this reflects a general property of Thr–Pro motifs or a context-specific outcome in CTD. Because CTR1 consists entirely of Thr–Pro pairs and displays natural sequence diversity across its repeats, it is an ideal system to test whether phosphorylation can induce proline isomerization in this context.

To detect proline isomerization, we employed proline-selective ^13^C direct-detect NMR experiments optimized to resolve overlapping proline resonances. Proline-selective experiments permit narrowing the sweep width, which improved the resolution of the proline peaks, and 3D experiments enabled straightforward *cis*/*trans* discrimination by comparing ^13^Cβ and ^13^Cγ shifts with chemical shift distributions present in the BMRB.^51^ Because proline-selective ^13^C,^15^N–CON spectroscopy was not available at the time of our prior Pol II CTD study, we collected control CTD spectra and confirmed that *cis*-Pro resonances were recorded (Figure S4). As expected for unphosphorylated SPT5 CTR1, no *cis*-Pro residues were present at detectable levels in the spectra (Figure 4). In contrast to the CTD results reviewed above, no additional resonances characteristic of *cis*-Pro were observed in phosphorylated CTR1. Instead, all observed proline chemical shifts remained consistent with a predominantly *trans* population, even under conditions in which multiple Thr–Pro sites were phosphorylated.

**Figure 4:**
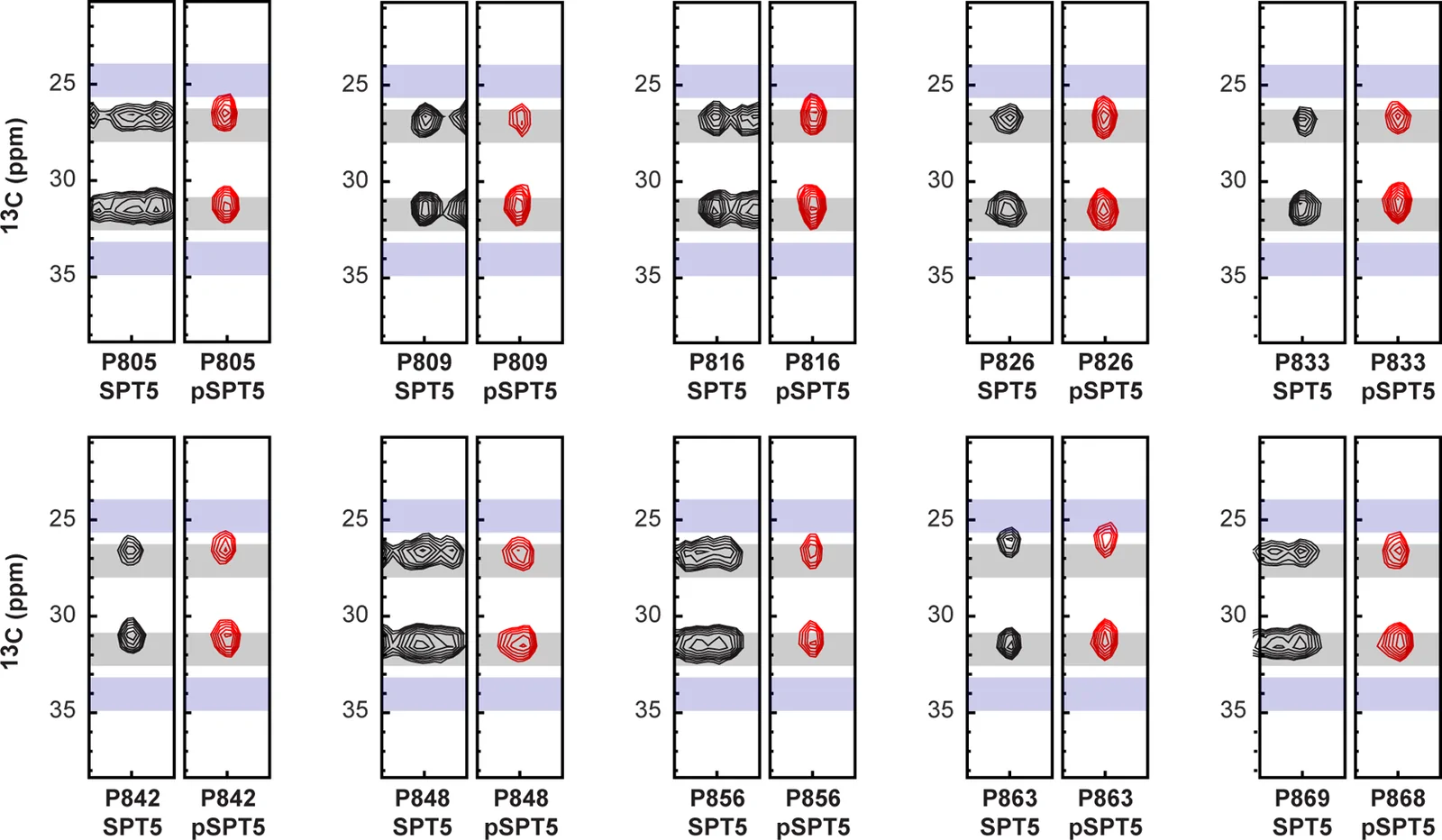
Threonine phosphorylation in the SPT5-CTR1 region does not drive a significant increase in the population of*cis*-proline. Strip plots from the proline-selective 3D (H)­CCCdNCO-IPAP spectrum correspond to unphosphorylated (black) and phosphorylated (red) CTR1. Gray bands indicate the expected range of chemical shifts for the ^13^Cβ and ^13^Cγ resonances of *trans-*proline side chains, while the blue bands represent the expectation values for the ^13^Cβ and ^13^Cγ resonances in the *cis-*proline state, as established by the BMRB.^51^

Although low-abundance *cis*-Pro conformers may exist below the detection limit, the contrast with CTD is striking. In CTD, *cis*-Pro signals are prominent and readily detected in 2D and 3D spectra following Ser5 phosphorylation, whereas in CTR1 they are entirely absent. These findings suggest that phosphorylation of Thr–Pro motifs in CTR1 does not yield *cis*-Pro in a population sufficient to be functionally relevant.

### Molecular Simulations Highlight Atomistic Differences in pSer and pThr

At the local structural level, the absence of phosphorylation-induced proline isomerization in CTR1 points to a fundamental distinction between Ser–Pro and Thr–Pro motifs. To evaluate the structural basis for proline isomerization in pSer-Pro but not pThr-Pro pairs, we used all-atom simulations of minimal peptides harboring Ser–Pro, pSer-Pro, Thr–Pro, or pThr-Pro motifs (GpX-P-G, where X is Ser or Thr).^28^ Previous studies of local conformations of pSer versus pThr suggest that pThr occupies a noncovalent pseudocyclized conformation that reflects a greater structural response to phosphorylation in Thr compared to Ser. This work established that the pThr-specific feature is driven by hydrogen bonding between the deprotonated phosphate oxygen(s) and the pThr amide proton.^53^ This difference likely arises from intrinsic chemical and conformational properties of pThr relative to pSer. In particular, the propensity of pThr to adopt a pseudocyclized conformation stabilized by intramolecular hydrogen bonding may restrict conformational flexibility and disfavor the backbone rearrangements required to stabilize *cis*-Pro. In contrast, pSer appears to retain greater conformational freedom, enabling sampling of positions that promote *cis*–*trans* isomerization. Together, these considerations support a model in which phosphorylation at threonine imposes local constraints that are incompatible with proline isomerization.

To assess atom proximity for potential hydrogen bonding, we measured the interatomic distances between the phosphorus of the phosphate moiety on either side chain and the amide proton of the phosphorylated residue. Importantly, this value will inherently be larger than a typical H-bond distance because we are measuring from the phosphorus instead of one of its deprotonated oxygens. The simulation-averaged P–HN distance is 4.36 Å for pSer versus 3.47 Å for pThr (Figure 5A), which reflects a conformation in which the phosphate moiety is up to 25% closer to the H-bond donor in pThr compared to pSer. Inspecting the H-bond geometry further using distance and angle cutoffs revealed that pThr peptides had 7–10× greater H-bond occupancy than pSer peptides (Figure S5). We reasoned that a pThr-specific hydrogen bond would lead to conformational restriction of the phosphate in pThr but not pSer. We calculated the RMSD of the phosphate group for each frame of the simulation (Figure 5B) and determined the average RMSD for each pSer (0.19 Å) and pThr (0.07 Å). Not only do the average RMSD values show less phosphate mobility in pThr, but the visual spread of trajectory RMSD values also indicates a larger distribution of accessible positions for pSer over pThr.

**Figure 5:**
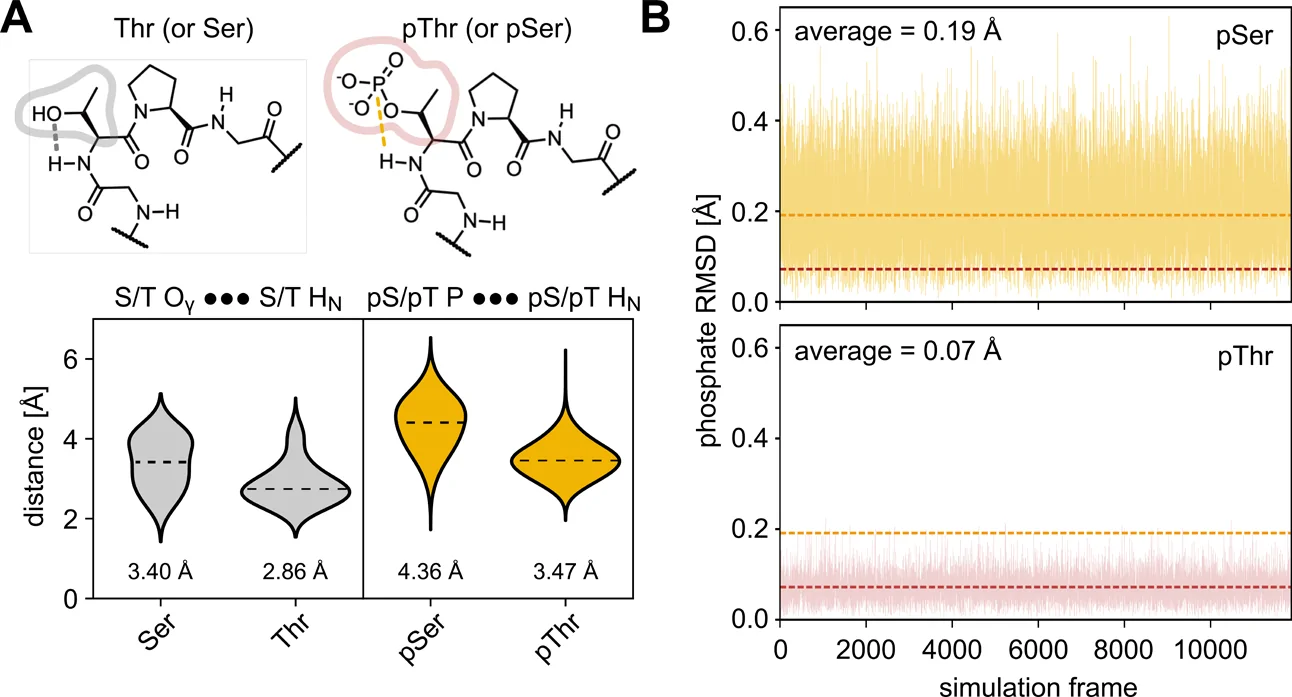
Phosphopeptide simulations reveal differential side chain motions in pSer and pThr. (A) Violin distributions of interatomic distances in short phosphopeptide Monte Carlo simulations of 11,875 frames each. The distances being measured are shown above the plot on the atomic structures of Ser/Thr or pSer/pThr peptides. Average interatomic distances across all frames are shown as dashed lines. Phosphorus is used as a proxy for the potential H-bond acceptor due to limitations in atom definitions. (B) Per-frame RMSD of the phosphate moiety (all six atoms of the phosphate) on pSer (top) or pThr (bottom). In the top panel, the yellow dashed line is the average RMSD of the pSer phosphate group over the simulation, while the red dashed line shows the pThr average for context. In the bottom panel, the red dashed line is the average RMSD of the pThr phosphate group with the average for pSer depicted as a yellow dashed line.

The lack of isomerization appears to reflect intrinsic differences between phosphorylated threonine and phosphorylated serine. Whereas Ser5 phosphorylation in CTD reliably shifts the *cis*/*trans* equilibrium, Thr-containing motifs in both CTD and CTR1 fail to do so. Although we know for pSer5 in the CTD heptads that the *cis*/*trans* proline population is influenced by the identity of the next residue in the primary structure, the simulations herein used minimal peptides of only four residues, which implies that the local conformation could be influenced by intrinsic differences in pSer and pThr chemistries alone. We may consider a model in which the backbone H-bond with the pThr phosphate induces local order that opposes the contacts that stabilize *cis*-Pro but cannot rule out a role for other interactions or local dynamics not captured here. Our results highlight that CTR1 phosphorylation does not trigger local proline isomerization, marking a mechanistic distinction from the CTD. We next asked whether phosphorylation alters CTR1 at the level of its overall conformational ensemble and therefore turned to SAXS.

### CTR1 Phosphorylation Does Not Affect Global Conformation

We next evaluated whether phosphorylation alters the global structural ensemble of CTR1. A common expectation, based on simple polyelectrolyte behavior, is that introducing multiple negative charges into an intrinsically disordered chain should promote expansion through electrostatic repulsion.^54,55^ Under this view, CTR1, which carries a modest net negative charge at neutral pH, might be predicted to expand upon phosphorylation. However, this assumption relies on treating the sequence as if it were dominated by one charge type (anions here). In contrast, many IDRs, including CTR1, contain a mixture of positive and negative charges, such that they behave as polyampholytes, where both the balance of positive and negative charges and their linear patterning strongly influence conformational dimensions.^56–58^

Studies on polyampholyte sequence physics demonstrate that chain dimensions are determined not simply by net charge, but by three key parameters: the fraction of charged residues (FCR), the net charge per residue (NCPR), and the patterning parameter kappa.^56^ When these parameters are applied to CTR1 using CIDER, both the unphosphorylated and phosphorylated sequences fall within the weak polyampholyte regime, where added negative charges are not predicted to drive substantial chain expansion (Figure S6), and feature significant mixing of positive and negative charge along the primary structure (Figure S7). The sequence descriptor kappa quantifies charge mixing across a protein sequence; CTR1 and phosphomimetic eCTR1 kappa values (0.2301 and 0.1570, respectively), suggest that the phosphorylation leads to a more even distribution of charges in the sequence. Thus, polyampholyte theory predicts that CTR1 should remain globally disordered with minimal change in overall dimensions upon phosphorylation, markedly different from the expansion expected under a simple polyelectrolyte model.

Our SAXS measurements align precisely with these predictions. Replicate scattering curves were highly consistent for both states (Figure 6A), while Guinier analysis confirmed the absence of aggregation (Figure 6B). Pair-distance distribution functions [P­(r)] overlapped closely between conditions, yielding *D*_max_ values of 105–120 Å and broadly similar distributions without evidence of expansion (Figure 6C). Radius of gyration (*R*_g_) values ranged from 28.0 to 29.9 Å across replicates, with no systematic difference between phosphorylation states (Table S2). Dimensionless Kratky plots from all four data sets overlapped almost perfectly and retained the characteristic monotonic rise of a disordered ensemble, with no indication of compaction or domain formation (Figure 6D). Together, these data indicate that CTR1 remains globally disordered and does not experience measurable expansion or compaction upon phosphorylation.

**Figure 6:**
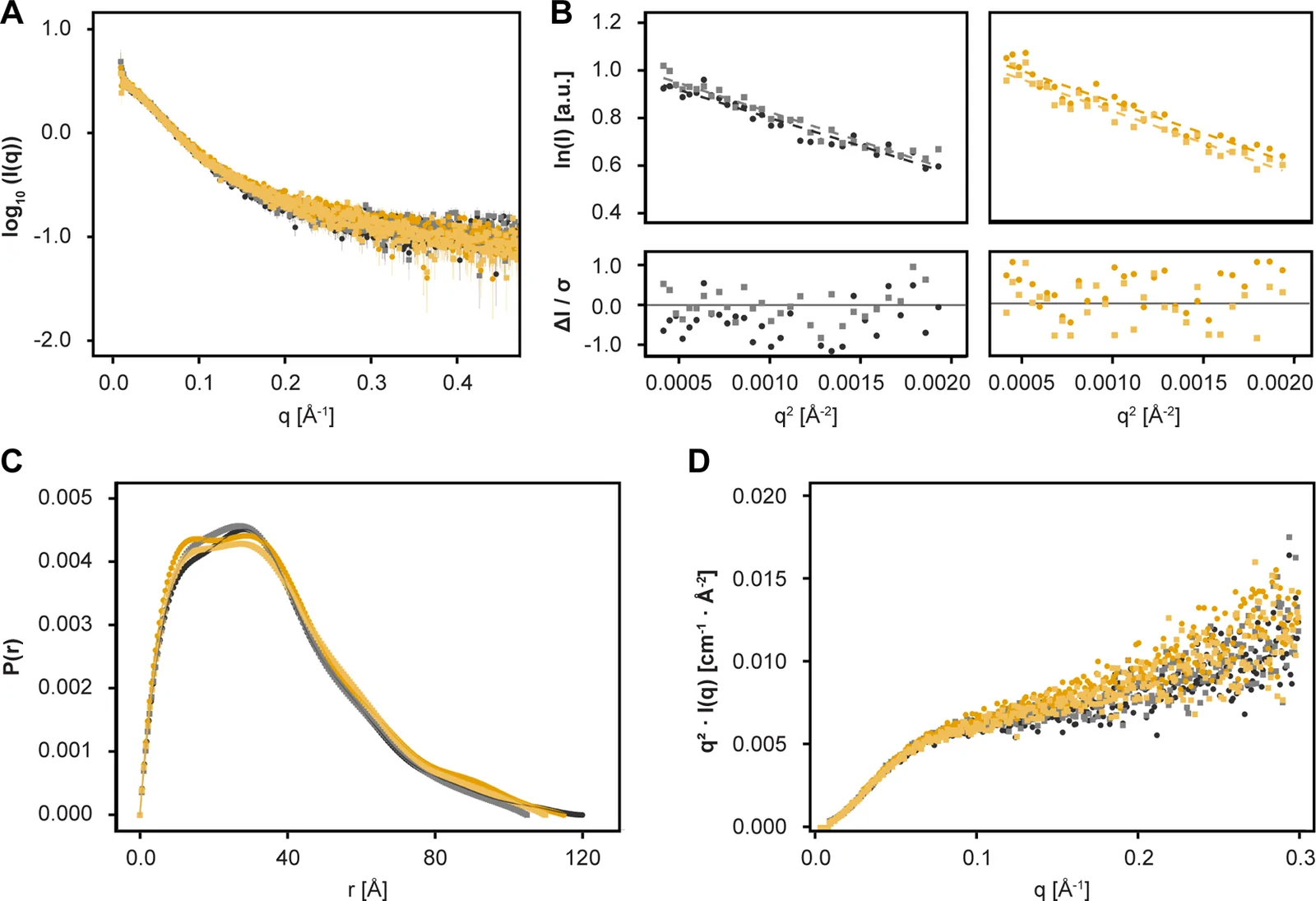
SAXS analysis reveals no change in the global dimensions of SPT5 CTR1 upon phosphorylation. (A) Scattering intensity *I*(*q*) vs *q* plots (log–log scale) demonstrate nearly identical scattering profiles for phosphorylated and unphosphorylated CTR1 across the entire q range. (B) Guinier analysis of unphosphorylated (gray) and phosphorylated (gold) CTR1 samples collected at 4 mg/mL. Linear fits (dashed lines) confirm the absence of aggregation in both states, and the residuals (bottom panels) show random scatter about zero. (C) Pair-distance distribution functions [P­(r)] derived from the scattering data overlap closely for both conditions, yielding *D*_max_ values of 105–120 Å and *R*_g_ values of 28–30 Å. (D) Dimensionless Kratky representations (*q* ^2^·*I*(*q*) vs *q*) reveal the characteristic monotonic rise of an intrinsically disordered ensemble, with no indication of compaction or domain formation.

Given the abundance of aromatic and proline residues in this sequence (∼9% and ∼11%, respectively), a tempting alternative explanation of our data is to speculate on “conformational buffering” through opposing intramolecular forces as the basis for the absence of conformational change upon multisite phosphorylation.^49,59^ In this way, the inherent sequence features of CTR1 could resist changes to the conformational ensemble upon phosphorylation. To compare a conformational buffering model and a weak polyampholyte model, we generated CTR1 ensemble predictions in STARLING with or without phosphomimetic glutamate residues at the experimental phospho-sites.^29^ Acknowledging the limitations of using phosphomimetics, we back-calculated SAXS scattering profiles from predictions to directly compare with SAXS experiments.^33^ The predicted and experimental profiles show good agreement (Figure 7A), which suggests that the predicted ensembles are globally similar in size and shape to the CTR1 constructs in vitro (Figure 6).^34^

**Figure 7:**
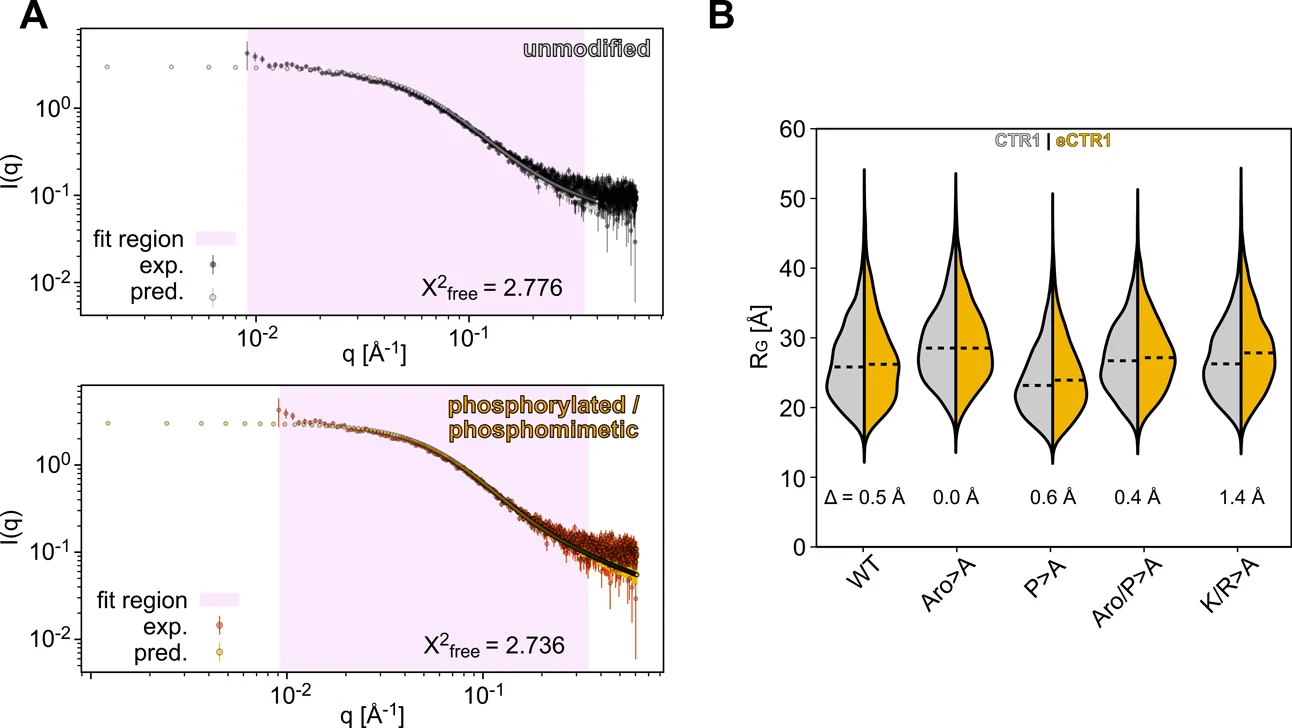
Global ensemble shape and size is captured by STARLING ensemble predictions. (A) SAXS profiles for unmodified CTR1 (top) and phosphorylated (bottom) compared to SAXS profiles calculated based on STARLING ensemble predictions of either CTR1 or phosphomimetic CTR1 (phosphothreonine > glutamate, eCTR1). The region for fitting/comparison is highlighted in pink. Visual inspection of these profiles shows good agreement between prediction and experiment, which is supported by relatively low values of the fitting statistic chi-squared free (Χ^2^_free_). (B) Radius of gyration distributions from ensemble predictions of 2000 conformers each for CTR1 (gray) and eCTR1 (gold) with various point substitutions (WT, wild-type; Aro > A, all aromatics to alanine; *P* > A, all prolines to alanine; Aro/*P* > A, all aromatics and prolines to alanine; K/R > A, all positive residues to alanine). The ensemble averaged radius of gyration (*R*_G_) is depicted as a horizontal dashed line. The difference between eCTR1 and CTR1 ensemble dimensions are shown below each violin.

The sticky aromatic residues (Tyr and Trp) are collectively associated with ensemble compaction and the rigid proline residues are associated with ensemble expansion (Figure 7B): In STARLING, mutating all aromatics to alanine increased the *R*_g_ by ∼2.5 Å, whereas mutating the proline residues to alanine decreased ensemble dimensions by ∼2.5 Å. Importantly, introducing negative charges to these sequences led to virtually no change in ensemble size, suggesting that while the aromatic and proline sequence biases determine ensemble dimensions, their presence is not the sole source of the observed conformational buffering. In line with the idea that the fraction of charged residues governs the global ensemble dimensions, ensembles with K/R to A mutations are indeed more sensitive to the addition of negative charges than the native sequence (Figure 7B). Together, these predicted ensembles support a model in which the positive residue content and patterning oppose a phosphorylation-linked conformational change in CTR1.

These findings mirror prior observations for the CTD, where SAXS similarly revealed unchanged global dimensions, but NMR detected local sequence-specific changes, such as proline isomerization.^19^ In contrast, our NMR analysis of CTR1 so far has not revealed local effects of phosphorylation; specifically, there is no evidence for proline isomerization. While this does not exclude the possibility of subtle or low-population structural biases, our current data suggest that CTR1 regulation does not involve global conformational changes.

Because CTR1 behaves as a weak polyampholyte, in which both positive and negative charges are distributed along the sequence, the chain dimensions are governed not only by net charge but also by charge patterning and intrachain interactions. Our results are therefore consistent with established polymer physics descriptions of IDRs, which predict that phosphorylation does not necessarily lead to global expansion. More broadly, these findings reinforce the importance of considering polyampholyte behavior when interpreting phosphorylation-dependent changes in intrinsically disordered proteins.

While phosphorylating multiple Thr–Pro motifs within CTR1 does not regulate function through a change in chain dimensions, it does raise the possibility of combinatorial or multivalent regulation of binding. Previous studies have shown that phosphorylated threonine residues in SPT5 can engage the Plus3 domain of RTF1, implicating CTR phosphorylation in establishment of transcriptional processivity.^60,61^ The presence of multiple phosphorylatable sites suggests several potential models: individual sites may function redundantly, distinct sites may encode different regulatory outcomes, or multiple sites may be engaged simultaneously to enable allovalent interactions. Although the highly phosphorylated states observed in vitro may not be fully populated in vivo, partially phosphorylated states containing multiple modified sites are likely to be accessible and could still support allovalent binding interactions. More broadly, the presence of multiple interaction sites within disordered regions has been widely observed and may represent a general strategy for tuning interaction strength and regulatory flexibility, with phosphorylation serving as a mechanism for generating or regulating these interactions.

### Tyrosine and Other Local Residues Modulate Phosphorylation Efficiency

Phosphorylation of intrinsically disordered proteins is often guided by sequence context, including residues flanking the modification site, and we have previously demonstrated that nearby tyrosine residues can help steer P-TEFb toward specific Ser–Pro sites in the context of RNA Pol II.^62^ To assess whether local sequence features modulate P-TEFb–dependent phosphorylation of SPT5 CTR1, we used site-directed mutagenesis to substitute specific residues within or adjacent to individual CTR1 repeats and analyzed the resulting modification patterns using MALDI-TOF mass spectrometry. Although this method is not formally quantitative, it enables direct comparison of phosphorylation outcomes under conditions designed to drive reactions to completion, using excess kinase and prolonged incubation. Under these conditions, relative changes in modification efficiency can reflect differences in substrate recognition.

Motivated by the observation that Tyr-1 in CTD heptads strongly enhances Ser-5 phosphorylation,^62^ we examined the role of tyrosine in CTR1 by substituting residues positioned analogously to the Tyr1–Ser5 spacing in the CTD. Although the SPT5 consensus pentamer G-S-R/Q-T-P does not specify an adjacent Tyr residue, they are common nearby the Thr–Pro pairs in CTR1 and so we substituted each with Ala to test their significance in determining P-TEFb substrate specificity. Only Y811A reduced phosphorylation (Figure 8B), whereas Y801A or Y843A had little to no effect (Figure S8). This reduction was consistently observed when Y811 was mutated in combination with other Tyr residues, underscoring the specificity of the observation to this position (for the full panel of MALDI traces, see Figure S8). Spacers between heptad motifs are rare in the Pol II CTD, and so a Tyr1 residue is also expected C-terminal to each Ser–Pro pair. Sequence inspection revealed that Y811 simultaneously aligns with both the equivalent of Tyr1 N-terminal to a Thr–Pro pair, and to the C-terminal Tyr1 position that would likely also exist in the sequence context of a CTD heptad, which may explain its disproportionate impact on phosphorylation efficiency. More broadly, these results highlight how sequence context can fine-tune P-TEFb substrate recognition, providing a framework for understanding how phosphorylation patterns are established in disordered regulatory regions.

**Figure 8:**
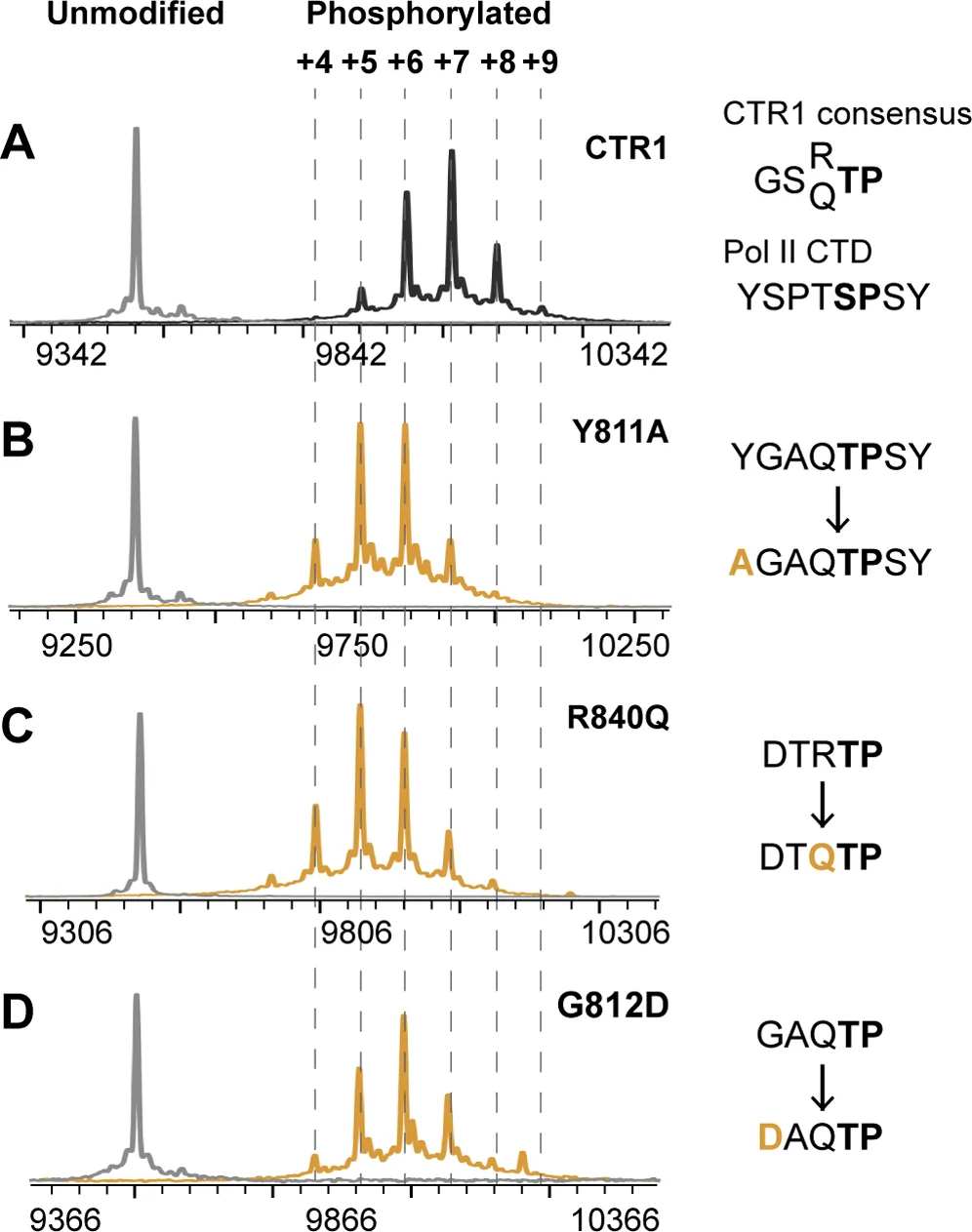
MALDI-TOF analysis reveals position-dependent effects of sequence context on CTR1 phosphorylation. (A) Representative mass spectrum of wild-type CTR1 showing the unmodified species (gray) and a distribution of phosphorylated states (black). The CTR consensus pentamer and CTD heptad repeat sequences are displayed to the left. (B–D) MALDI-TOF spectra of representative mutants illustrating the effects of sequence perturbations on phosphorylation for (B) tyrosine substitution through Y811A, (C) arginine substitution through R840Q, and (D) glycine substitution through G812D. In all the mutagenesis panels, unmodified species are shown in gray and phosphorylated species in gold. Dashed lines and labels denote phosphorylation states and are included as visual guides to facilitate peak assignment. Right panels depict the corresponding sequence context for each construct, with mutations highlighted and aligned relative to the CTR1 consensus or the Pol II CTD motif.

Beyond tyrosine, other conserved positions within the CTR1 consensus sequence (G–S–R/Q–T–P) appeared likely to influence phosphorylation efficiency. We examined the role of arginine at position three of the repeat. Notably, the three CTR1 repeats that were not phosphorylated all contained arginine at this position, prompting us to test whether substituting arginine with glutamine would enhance modification (Figures 8C and S9). Contrary to expectation, individual R → Q substitutions reduced phosphorylation, as demonstrated by R840Q (Figure 8C), and the effect was more pronounced when all three positions were mutated simultaneously (Figure S9). This outcome suggests that arginine at this position plays a more complex role than anticipated. While these glutamine substitutions remove a positive charge, they also introduce a polar side chain capable of forming hydrogen bonds, raising the possibility that altered local interactions rather than charge alone underlie the reduced phosphorylation. Consistent with the idea, exploratory SAXS measurements of the triple R → Q variant showed non-native scattering behavior indicative of self-association or aggregation under conditions where wild-type CTR1 remained monomeric and well behaved (data not shown). The results support the conclusion that glutamine substitution perturbs the physical properties of CTR1 in a manner distinct from arginine removal alone. Together, these findings indicate that arginine at this position contributes to phosphorylation efficiency and that its substitution with glutamine introduces additional interactions that are unfavorable for P-TEFb-mediated modification.

We also evaluated the role of glycine at the first position of the repeat, since nearly all phosphorylated repeats contained glycine, whereas unmodified repeats contained bulkier residues. To test whether glycine was required, we mutated G812 within a phosphorylated repeat to aspartate, which diminished phosphorylation (Figure 8D). Conversely, introducing glycine into an unphosphorylated repeat (D838G), or replacing it with alanine or leucine (D838A, D838L), had little effect on phosphorylation status (Figure S10). These findings indicate that glycine in the first pentamer position is not strictly required for phosphorylation by P-TEFb but may play a permissive role, supporting modification when present without being sufficient to drive it on its own.

Together, these mutagenesis experiments demonstrate that P-TEFb substrate selection on CTR1 is influenced by more than the core TP motif. Tyrosine exerts the strongest positional effect, enhancing phosphorylation when located three residues upstream of the TP motif, while arginine and glycine play subtler, context-dependent roles. Arginine appears to contribute through its positive charge, whereas glycine provides a permissive but not essential environment for modification. Our end point-based MALDI analysis highlights how flanking residues tune phosphorylation efficiency and shape the modification landscape across CTR1 repeats.

## Conclusions

In this study, we investigated how threonine phosphorylation shapes the structural properties of the SPT5 CTR1 domain, an intrinsically disordered repeat region central to transcriptional elongation control. By integrating NMR spectroscopy, SAXS, mass spectrometry, and all-atom and coarse-grained ensembles, we show that P-TEFb phosphorylates CTR1 in a selective and heterogeneous manner, modifying a subset of Thr–Pro motifs without inducing local proline isomerization or global conformational rearrangement. These findings stand in contrast to prior observations for serine-containing repeats, where phosphorylation can drive local structural effects, detectable through downstream interactions, despite similarly unchanged global dimensions. Together, our results demonstrate that phosphorylation at threonine and serine residues can have fundamentally different structural consequences, even within closely related repeat architectures.

Importantly, our data indicate that the absence of structural response in CTR1 is not an anomalous feature of this construct but instead reflects broader principles governing intrinsically disordered proteins. Polyampholyte-based sequence analysis correctly predicts the lack of phosphorylation-induced expansion, highlighting the limitations of applying simple polyelectrolyte intuition to disordered regulatory domains. Mutagenesis further reveals that residues flanking the Thr–Pro motif—particularly tyrosine at defined spacings—bias kinase recognition and contribute to selective modification, while other conserved positions exert context-dependent effects. Collectively, these observations support a model in which threonine phosphorylation functions primarily as a regulatory signal rather than a structural switch. Instead of driving large-scale conformational changes, it likely encodes specificity through sequence context to facilitate discrete partner interactions, such as the recruitment of the RTF1 Plus3 domain to establish transcriptional processivity. More broadly, this work underscores that seemingly conservative substitutions between serine and threonine can have unexpected consequences in disordered systems, with implications for how phosphorylation is interpreted across transcriptional regulatory networks.

## Supplementary Material



## Notes

Two tables of STARLING simulation sequence parameters and SAXS analysis parameters. Nine figures providing chemical shift perturbations, full sweep-width spectra, control spectra for RNA polymerase II CTD, charge-based sequence analysis, and mass spectrometry data for all mutants (PDF)
