# Supplementary material for: Multisite Threonine Phosphorylation in the SPT5 C‑Terminal Region 1 Enables Sequence-Dependent Regulation without Global Structural Remodeling
Source: J Phys Chem B. 2026 Aug 10;130(33):8409–22. doi: 10.1021/acs.jpcb.6c03886 (PMC13557439; doi:10.1021/acs.jpcb.6c03886)
Supplement: Supplementary file 1 [file jp6c03886_si_001.pdf]

Supporting Information

Multisite threonine phosphorylation in the SPT5 C-terminal region 1 enables sequence-dependent regulation without global structural remodeling

Rong Hu[1], Emery T. Usher[2], Olivia A. Fraser[3], Wei Chen[1,3], Alex S. Holehouse[4,5], and Scott A. Showalter[1,3]*

[1]Department of Chemistry, The Pennsylvania State University, University Park, PA 16802, United States

[2]Department of Chemistry and Biochemistry, University of California, Santa Cruz, CA 95064, United States

[3]Center for Eukaryotic Gene Regulation, Department of Biochemistry and Molecular Biology, The Pennsylvania State University, University Park, PA 16802, United States

[4]Department of Biochemistry and Molecular Biophysics, Washington University School of Medicine, St. Louis, MO 63110, United States

[5]Center for Biomolecular Condensates, Washington University in St. Louis, St. Louis, MO 63110, United States

Table S1. STARLING sequence and ensemble parameters.

name	sequence	NCPR	FCR	kappa	R_g (Å)
CTR1	SNAGSVSTYGRTPARTPGYGAQTPSYT AAGSKTPLVGSQTPNWDTDTRTPYGTM TPSHDGSMTPRHGAWDPTANTTPARNN DFDYSLEE	-0.0067	0.1416	0.2301	26.5
eCTR1	SNAGSVSTYGRTPARTPGYGAQEPSYT AAGSKEPLVGSQEPNWDTDTRTPYGTM EPSHDGSMEPRHGAWDPTANTEPARNN DFDYSLEE	-0.0742	0.2090	0.1570	27.0
CTR1 Aro>A	SNAGSVSTAGRTPARTPGAGAQTPSAT AAGSKTPLVGSQTPNADTDTRTPAGTM TPSHDGSMTPRHGAADPTANTTPARNN DFDASLEE	-0.0067	0.1416	0.2301	29.1
eCTR1 Aro>A	SNAGSVSTAGRTPARTPGAGAQEPSAT AAGSKEPLVGSQEPNADTDTRTPAGTM EPSHDGSMEPRHGAADPTANTEPARNN DFDASLEE	-0.0742	0.2090	0.1570	29.1
CTR1 P>A	SNAGSVSTYGRTAARTAGYGAQTASYT AAGSKTALVGSQTANWDTDTRTAYGT MTASHDGSMTARHGAWDATANTTAA RNNDFDYSLEE	-0.0067	0.1416	0.2301	24.0
eCTR1 P>A	SNAGSVSTYGRTAARTAGYGAQEASYT AAGSKEALVGSQEANWDTDTRTAYGT MEASHDGSMEARHGAWDATANTEAA RNNDFDYSLEE	-0.0742	0.2090	0.1570	24.6
CTR1 Aro/P>A	SNAGSVSTAGRTAARTAGAGAQTASAT AAGSKTALVGSQTANADTDTRTAAGT MTASHDGSMTARHGAADATANTTAAR NNDFDASLEE	-0.0067	0.1416	0.2301	27.2
eCTR1 Aro/P>A	SNAGSVSTAGRTAARTAGAGAQEASAT AAGSKEALVGSQEANADTDTRTAAGT MEASHDGSMEARHGAADATANTEAAR NNDFDASLEE	-0.0742	0.2090	0.1570	27.6
CTR1 K/R>A	SNAGSVSTYGATPAATPGYGAQTPSYT AAGSATPLVGSQTPNWDTDTATPYGT MTPSHDGSMTPAHGAWDPTANTTPAA NNDFDYSLEE	-0.0742	0.0742	0.2485	27.0
eCTR1 K/R>A	SNAGSVSTYGATPAATPGYGAQEPSYT AAGSAEPLVGSQEPNWDTDTATPYGT MEPSHDGSMEPAHGAWDPTANTEPAA NNDFDYSLEE	-0.1416	0.1416	0.1393	28.4

Table S2. SAXS analysis on unphosphorylated and phosphorylated CTR1

**Unphosphorylated CTR1**						
Replicate	R_g (Å) Guinier	Range (points)	I(0)	R_g (Å) from P(r)	Range (points)	D_{max} (Å)
1	29.46 ± 0.90	20-43	2.94 ± 0.049	29.13 ± 2.89	22-300	120
2	28.16 ± 0.57	22-47	2.95 ± 0.035	28.25 ± 2.92	20-300	105
**Phosphorylated CTR1**						
Replicate	R_g (Å) Guinier	Range (points)	I(0)	R_g (Å) from P(r)	Range (points)	D_{max} (Å)
1	29.71 ± 0.94	20-46	3.17 ± 0.061	29.66 ± 3.09	19-280	115
2	29.33 ± 0.84	20-47	3.04 ± 0.053	29.59 ± 2.97	20-300	110

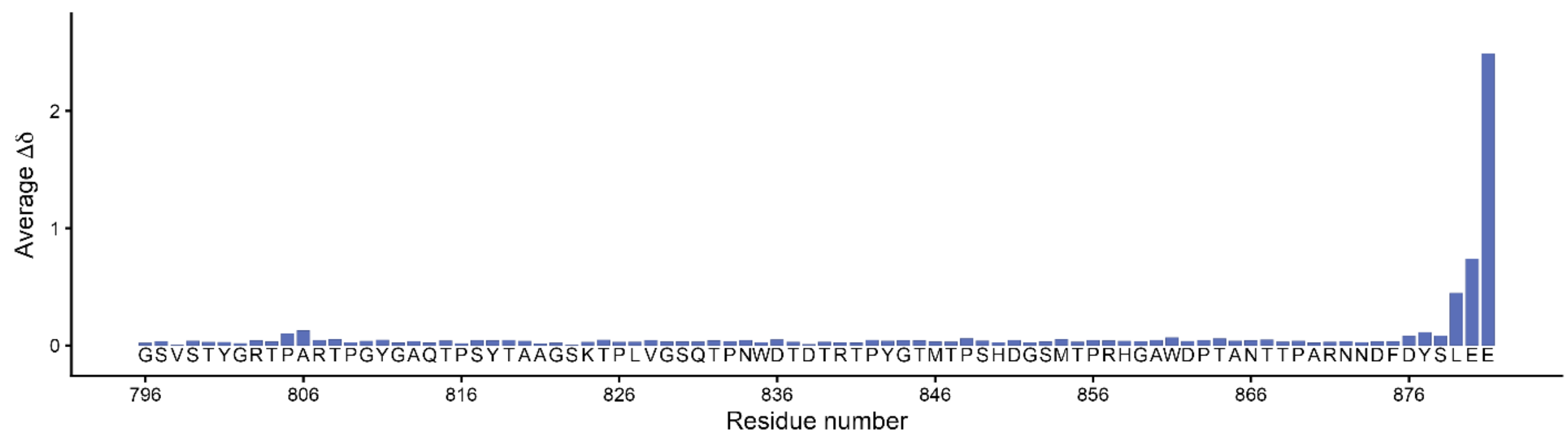

Figure S1: NMR chemical shift perturbations for SPT5-CTR1 relative to full-length SPT5-CTR. Chemical shift perturbations are small and random throughout the chain, apart from the final ~6 residues located at the newly introduced C-terminus. Overall, these data suggest that the global conformation of the CTR1 region is unchanged, relative to its conformation in full-length CTR.

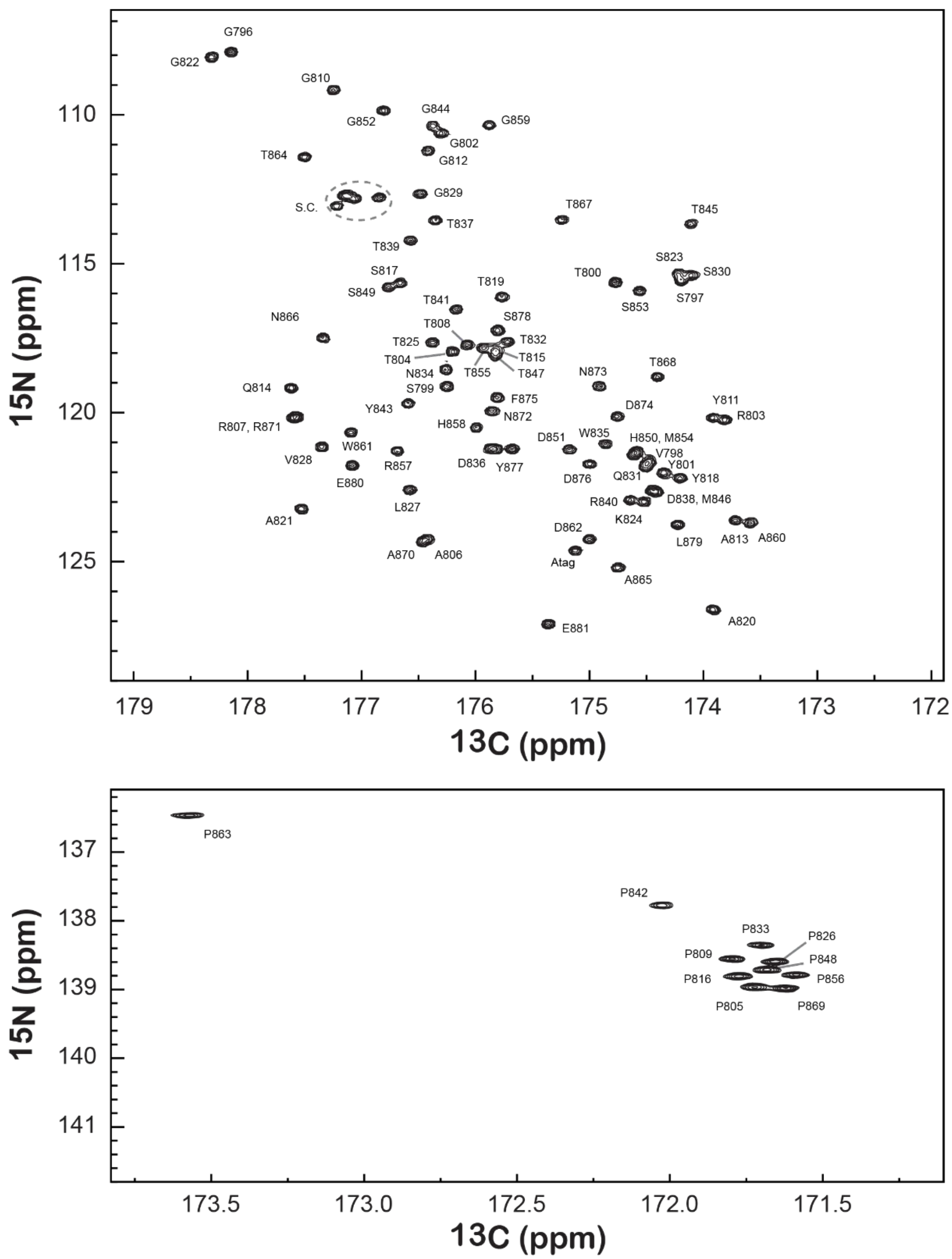

Figure S2: Annotated ^{13}C,^{15}N-CON spectra of unphosphorylated SPT5-CTR1. (A) ^{13}C,^{15}N-CON displaying the resonances for all residues except those for which a proline contributes the ^{15}N to the observed correlation. Resonances from asparagine side chains (S.C.) are enclosed in a dashed circle. (B) Proline-selective ^{13}C,^{15}N-CON displaying these resonances with the improved resolution afforded by the selective spectrum. All resonances are labeled with assignments indicating the amino acid type and associated residue number within full-length SPT5, where the residue contributing the ^{15}N to the correlation is used for naming.

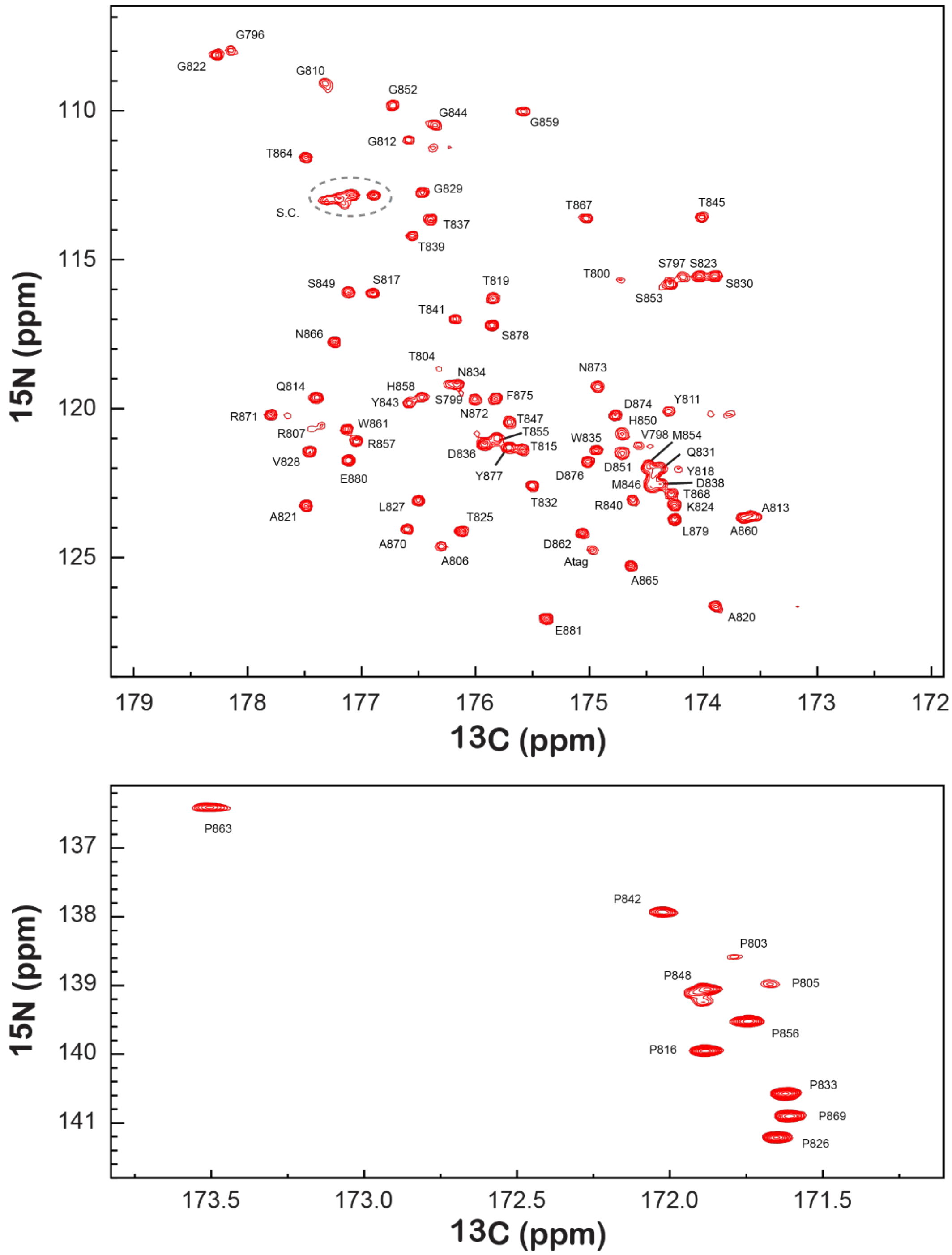

Figure S3: Annotated ^{13}C,^{15}N-CON spectra of phosphorylated SPT5-CTR1. (A) ^{13}C,^{15}N-CON displaying the resonances for all residues except those for which a proline contributes the ^{15}N to the observed correlation. Resonances from asparagine side chains (S.C.) are enclosed in a dashed circle. (B) Proline-selective ^{13}C,^{15}N-CON displaying these resonances with the improved resolution afforded by the selective spectrum. All resonances are labeled with assignments indicating the amino acid type and associated residue number within full-length SPT5, where the residue contributing the ^{15}N to the correlation is used for naming.

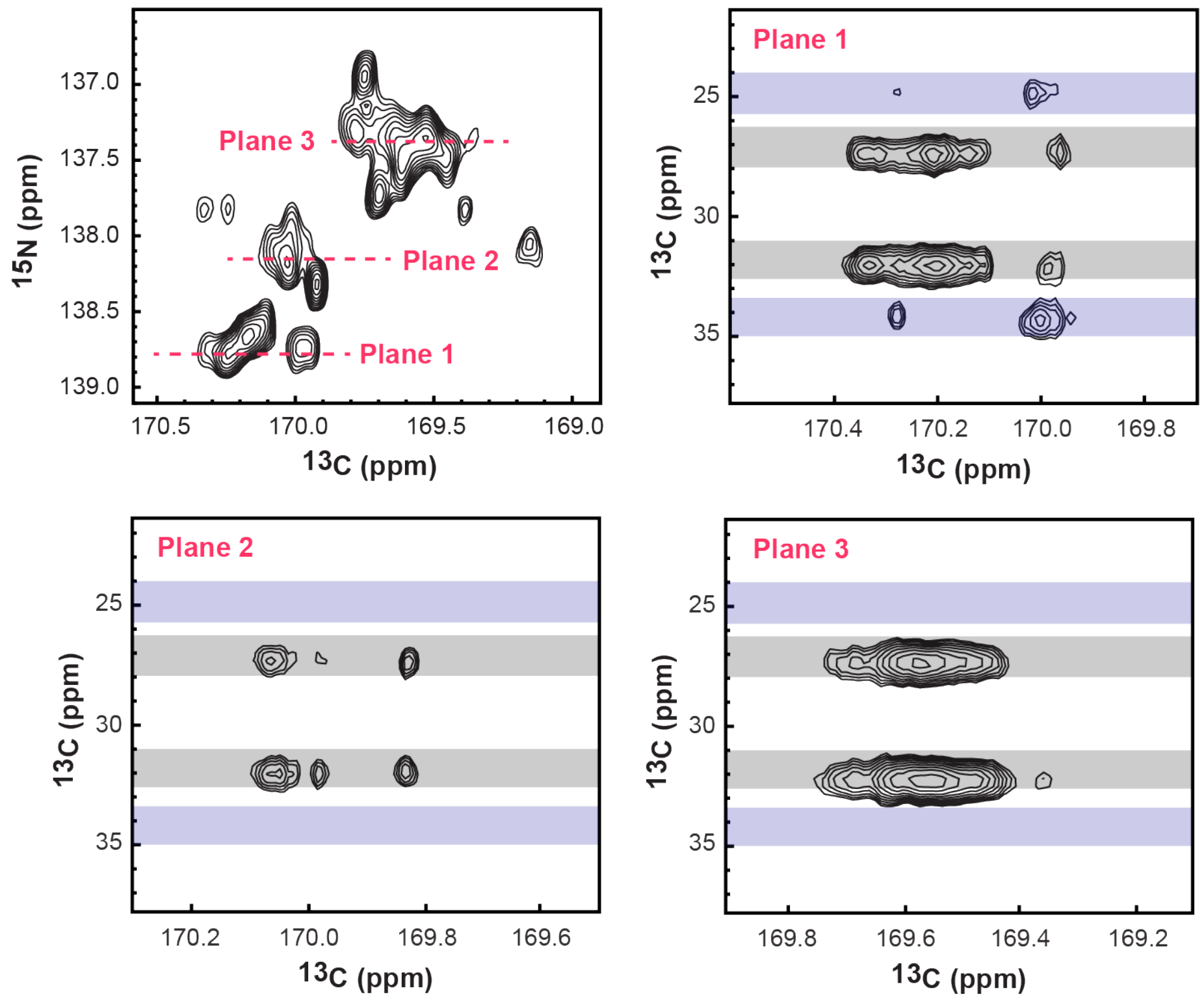

Figure S4: Evidence of high-population *cis*-proline resonances in the pSer5-enriched state of RNA Polymerase II C-terminal Domain. (A) The ^{13}C,^{15}N-CON spectrum of phosphorylated CTD, zoomed to display resonances where the nitrogen is contributed by proline residues. Dotted red lines labeled as Plane 1, Plane 2, and Plane 3 indicate the ^{15}N chemical shift at which planes were selected from the 3D (H)CCCdNCO-IPAP for display in the remaining panels. (B) Plane 1 contains a mixture of proline spin systems in which the ^{13}Cβ and ^{13}Cγ resonances are consistent with *trans*-proline and those which are consistent with *cis*-proline. (C, D) All spin systems in these two selected planes contain ^{13}Cβ and ^{13}Cγ resonances consistent with the *trans*-proline state. In all panels, grey bands indicate the expected range for *trans*-proline and blue bands for *cis*-proline based on distributions in the BioMagResBank.(51)

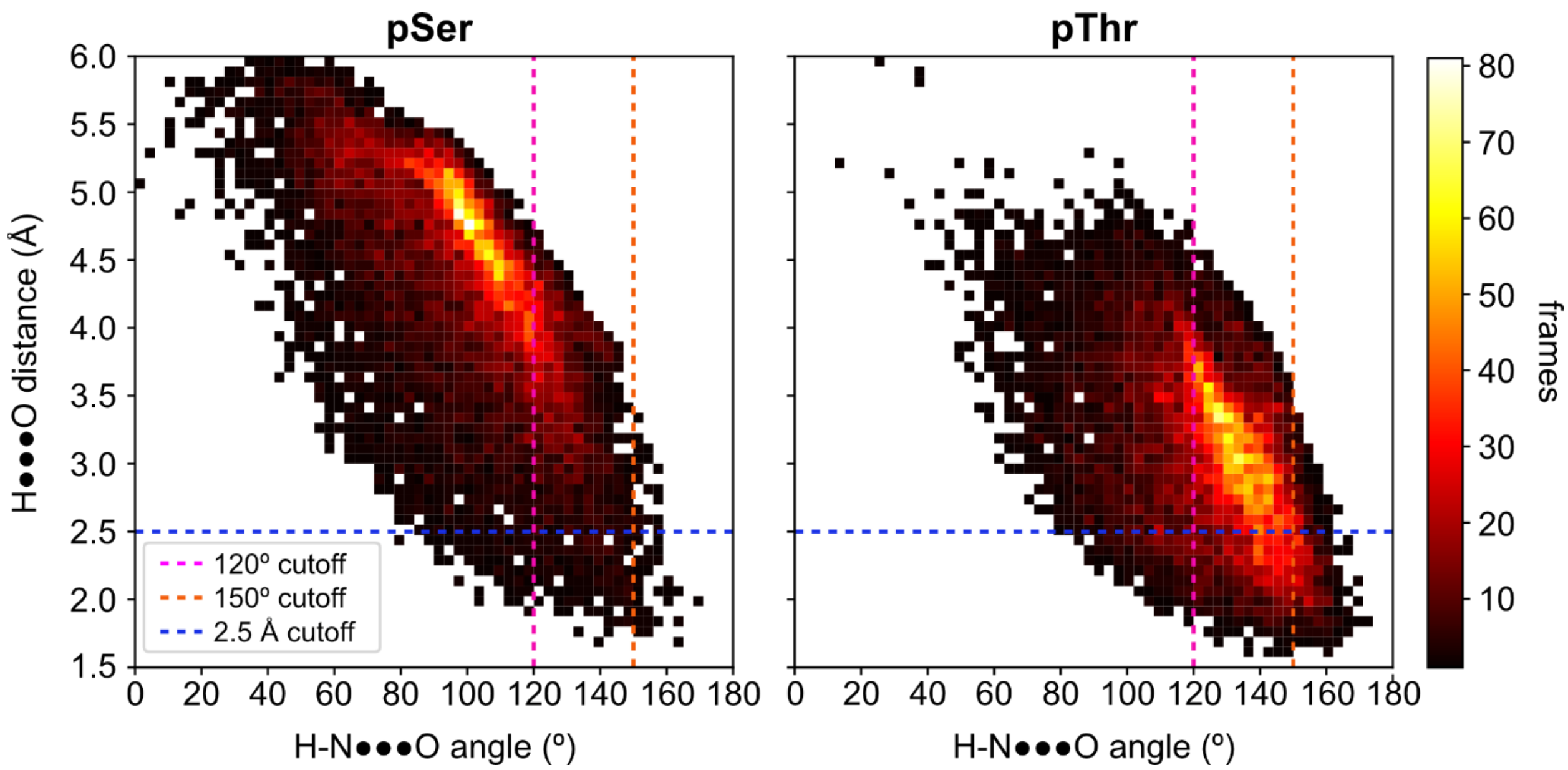

Figure S5: Intra-residue hydrogen bond occupancy in pThr is significantly greater than that of pSer. Per-frame distances and angles were calculated from simulations of pSer and pThr peptides with 11,875 frames each. The distance cutoff for a 'true' H-bond was set to 2.5 Å and the H-bond angle was considered with a strict (150°) or relaxed (120°) cutoff. Using these metrics, we calculated the H-bond occupancy for pSer (left) and pThr (right). The pSer H-bond occupancy (presented as percentage of simulation frames) was between 0.38 (strict) and 2.53 (relaxed) percent. The pThr H-bond occupancy was between 4.61 (strict) and 16.70 (relaxed) percent. With the strict cutoff, pThr is 7.35x more likely to satisfy the H-bond criteria than pSer ($p << 0.001$, Fisher's exact test, n=11875). With the relaxed cutoff, pThr is 10.95x more likely than pSer to satisfy the H-bond criteria ($p << 0.001$, Fisher's exact test, n=11875).

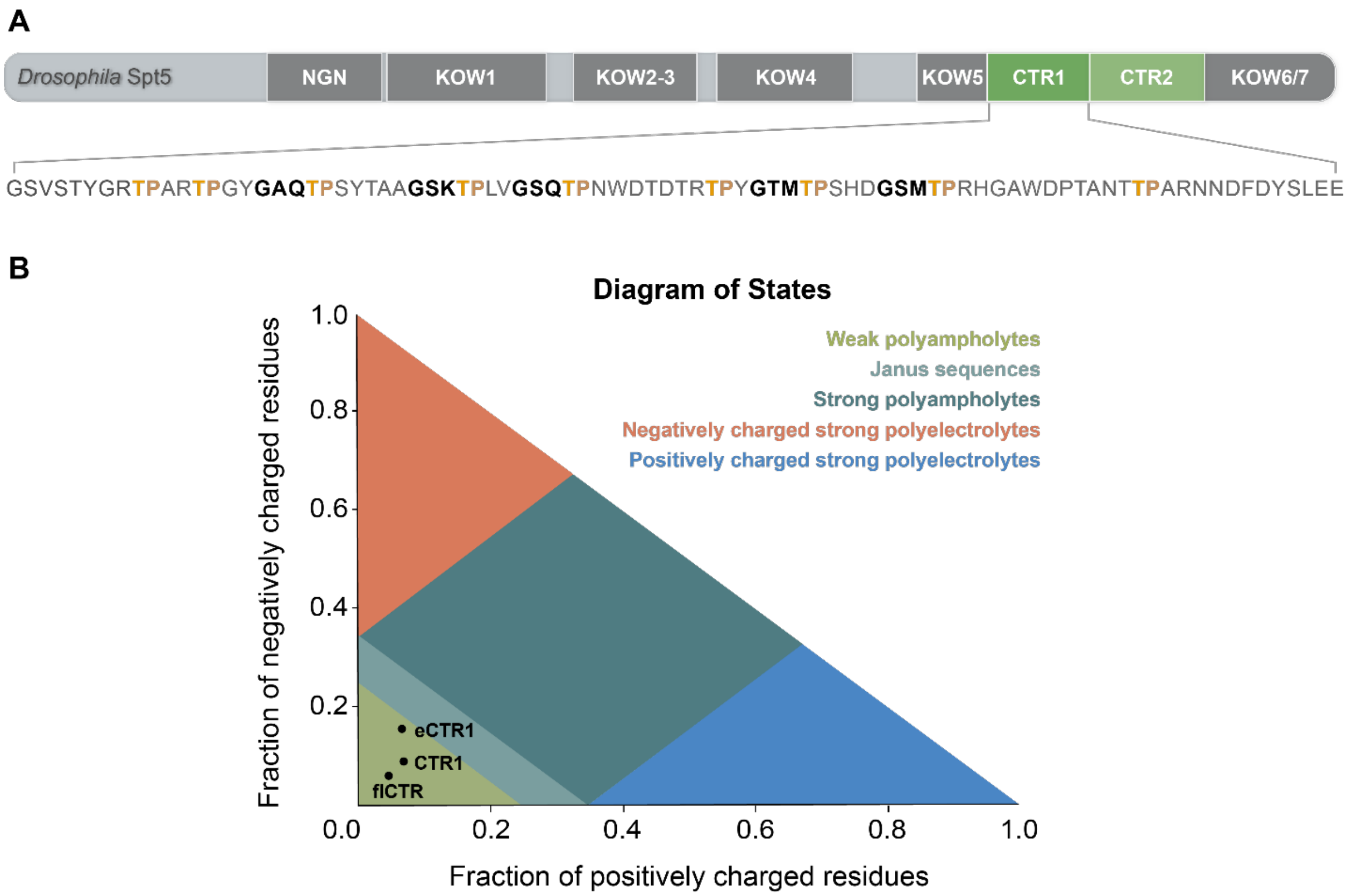

Figure S6: Charge-based analysis of SPT5 CTR sequences. (A) Schematic representation of *Drosophila* SPT5 domain organization, highlighting the C-terminal repeat regions (CTR1 and CTR2). (B) Placement of SPT5 CTR constructs on the charge-based diagram of states, based on parameters obtained from CIDER analysis. CTR1 and flCTR map to the weak polyampholyte regime, indicating low net charge per residue and a relatively well-mixed charge distribution.

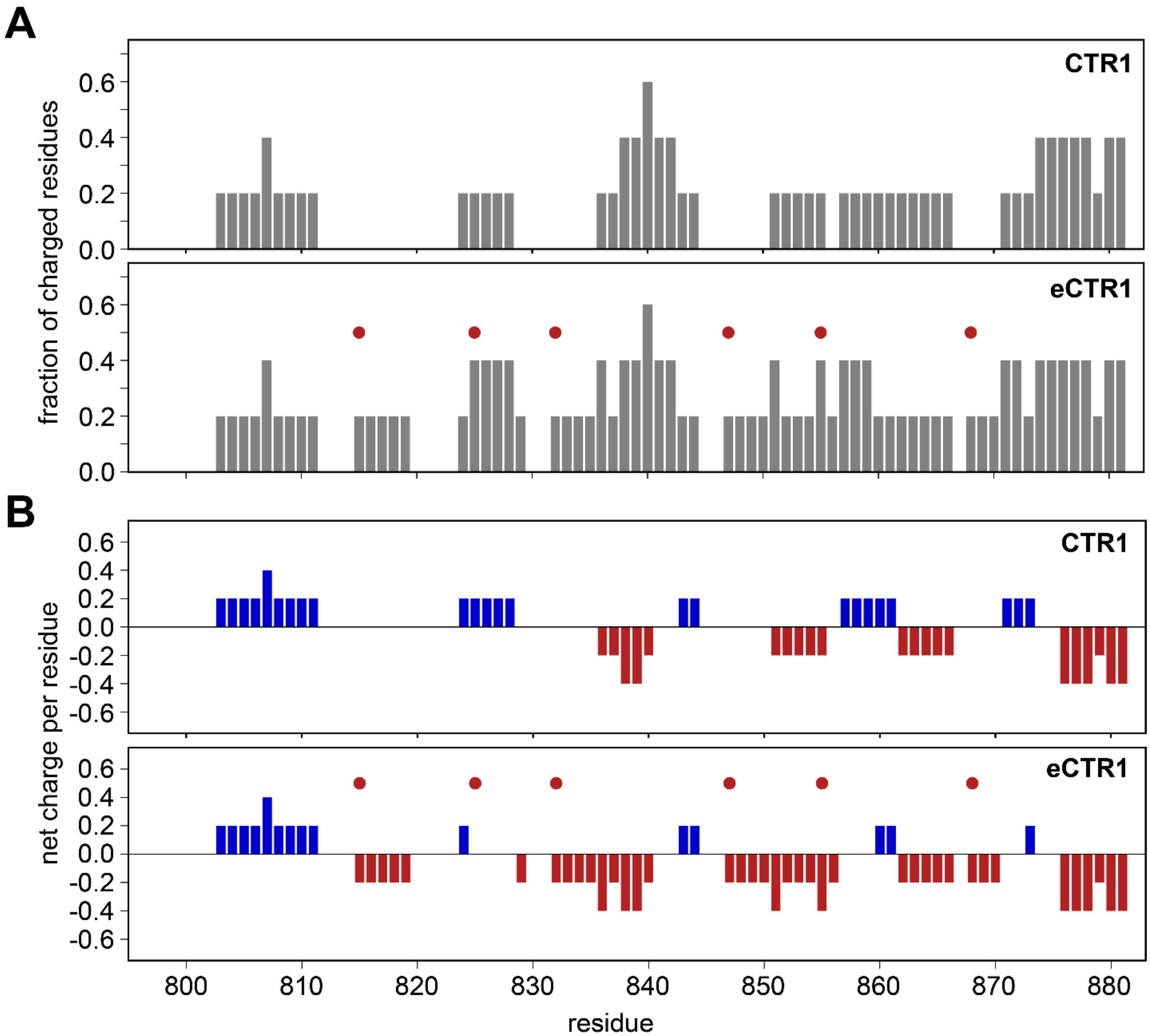

Figure S7: CTR1 linear sequence features inform likely ensemble behavior. (A) Linear profile showing the fraction of charged residues (FCR) at neutral pH in unmodified (top, average FCR = 0.1416) and hyperphosphorylated (bottom, average FCR = 0.2090) CTR1 using Glu to mimic negative charge of the phospho-state. Based on IDR averages for the human proteome, FCR < 0.1 is highly depleted in charged residues, whereas FCR > 0.3 would be highly enriched, meaning that neither CTR1 nor its hyperphosphorylated form are notable in terms of their charge content. Importantly, the linear FCR plots show that the charged residues are well distributed throughout the chain. (B) Linear profile of net charge per residue (NCPR) for CTR1 (top, average NCPR = -0.0067) and eCTR1 (bottom, average NCPR = -0.0742). Values hovering around zero (-0.1 to +0.1) indicate a relative balance of positive and negative residues across the chain. In both panels, MS-validated phosphorylation sites are denoted by red circles.

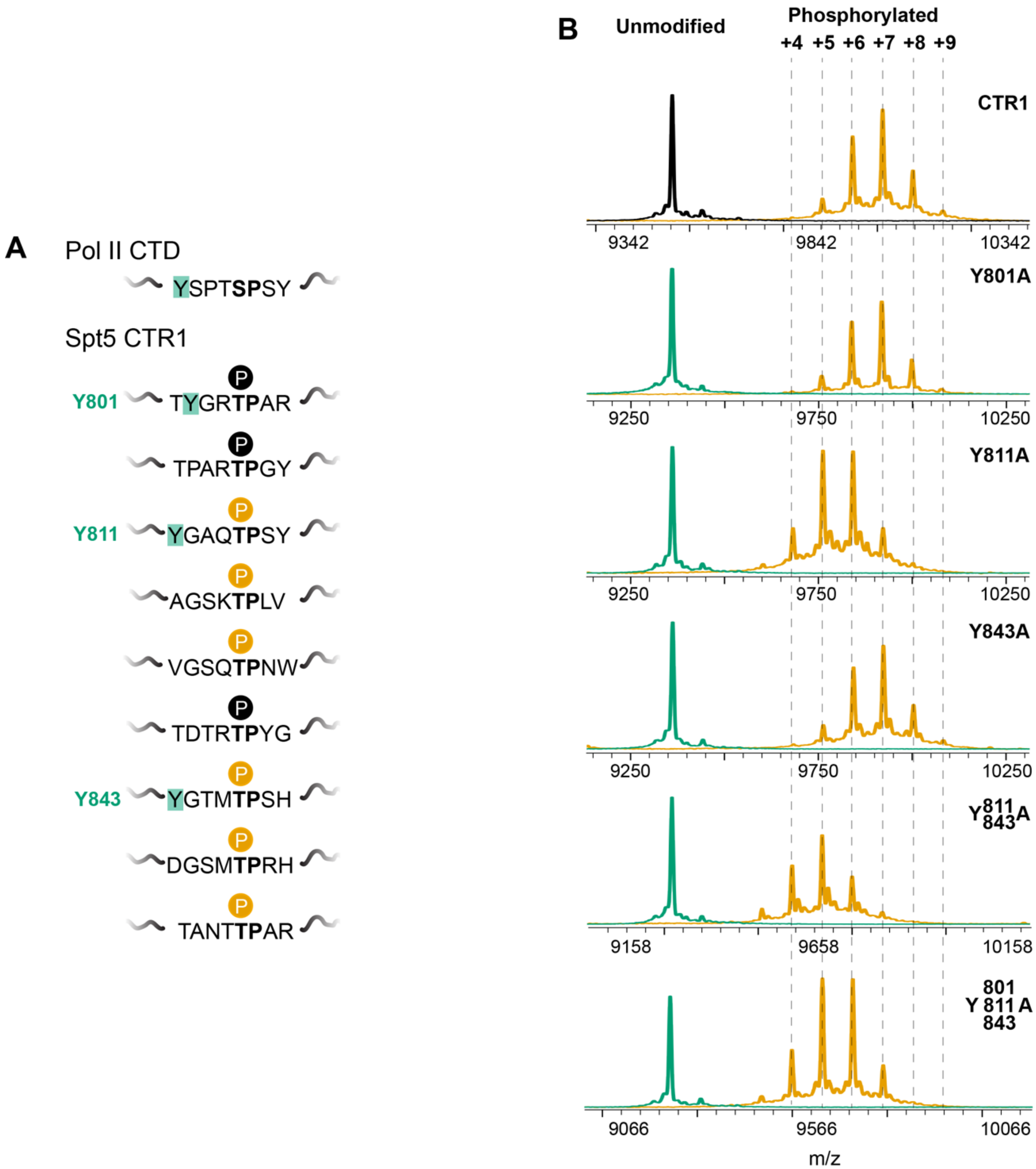

Figure S8: Sequence context influences the extent of CTR1 phosphorylation by P-TEFb (Tyr substitutions). (A) Schematic representation of the SPT5 CTR1 region highlighting individual pentapeptide repeats containing Thr–Pro motifs. Residues targeted for mutagenesis are indicated. Phosphorylated threonine residues identified by NMR are marked with circled orange "P" symbols. (B) MALDI-TOF mass spectra of unmodified and phosphorylated wild-type and Tyr-substitution mutant CTR1 constructs. In each spectrum, unmodified species are shown in black (wild type) or green (Tyr mutants), and phosphorylated species are shown in gold. Dashed lines and labels denote phosphorylation states and are included as visual guides to facilitate peak assignment.

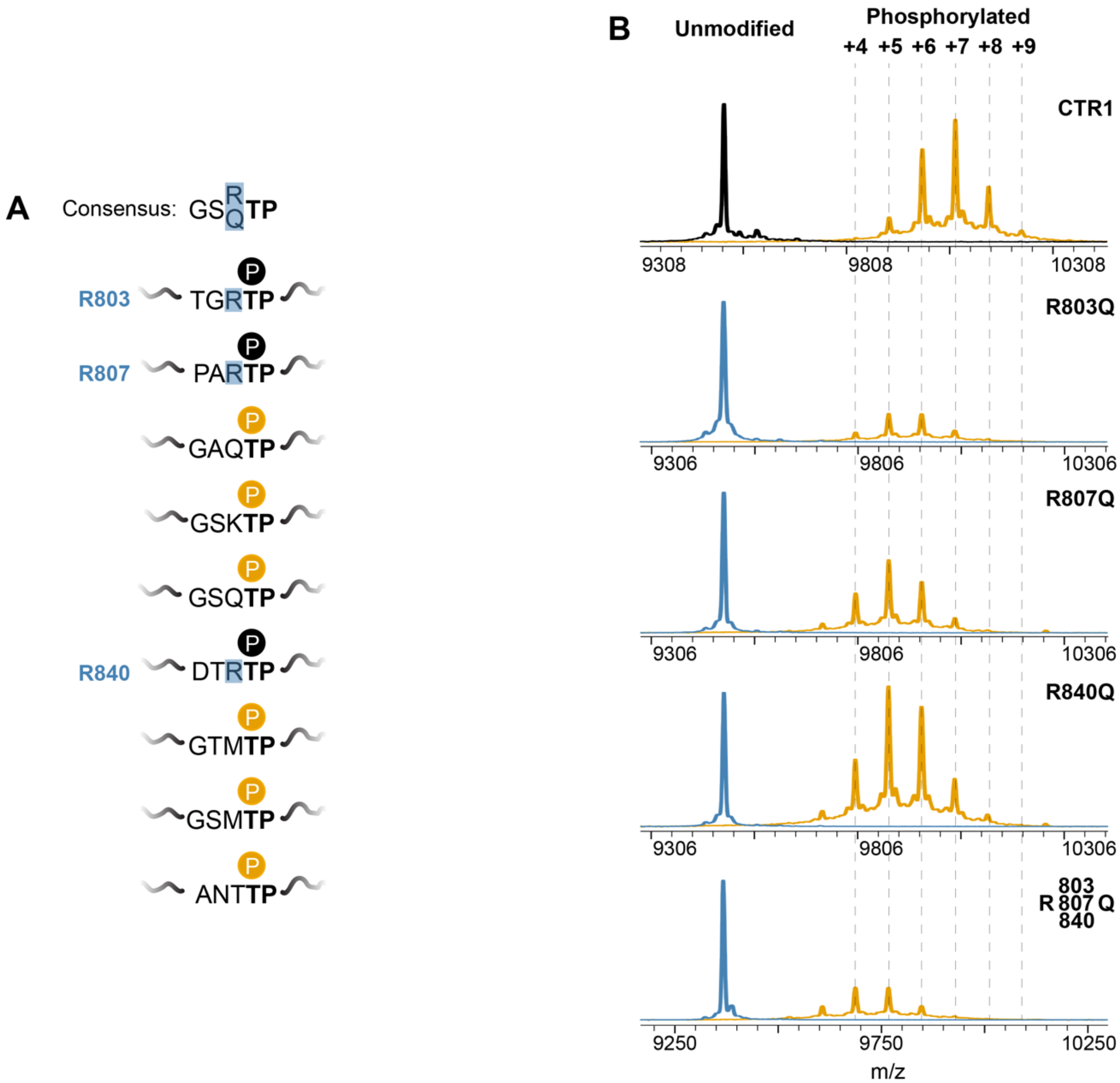

Figure S9: Sequence context influences the extent of CTR1 phosphorylation by P-TEFb (Arg substitutions). (A) Schematic representation of the SPT5 CTR1 region highlighting individual pentapeptide repeats containing Thr–Pro motifs. Residues targeted for mutagenesis are indicated. Phosphorylated threonine residues identified by NMR are marked with circled orange "P" symbols. (B) MALDI-TOF mass spectra of unmodified and phosphorylated wild-type and Arg-substitution mutant CTR1 constructs. In each spectrum, unmodified species are shown in black (wild type) or blue (Arg mutants), and phosphorylated species are shown in gold. Dashed lines and labels denote phosphorylation states and are included as visual guides to facilitate peak assignment.

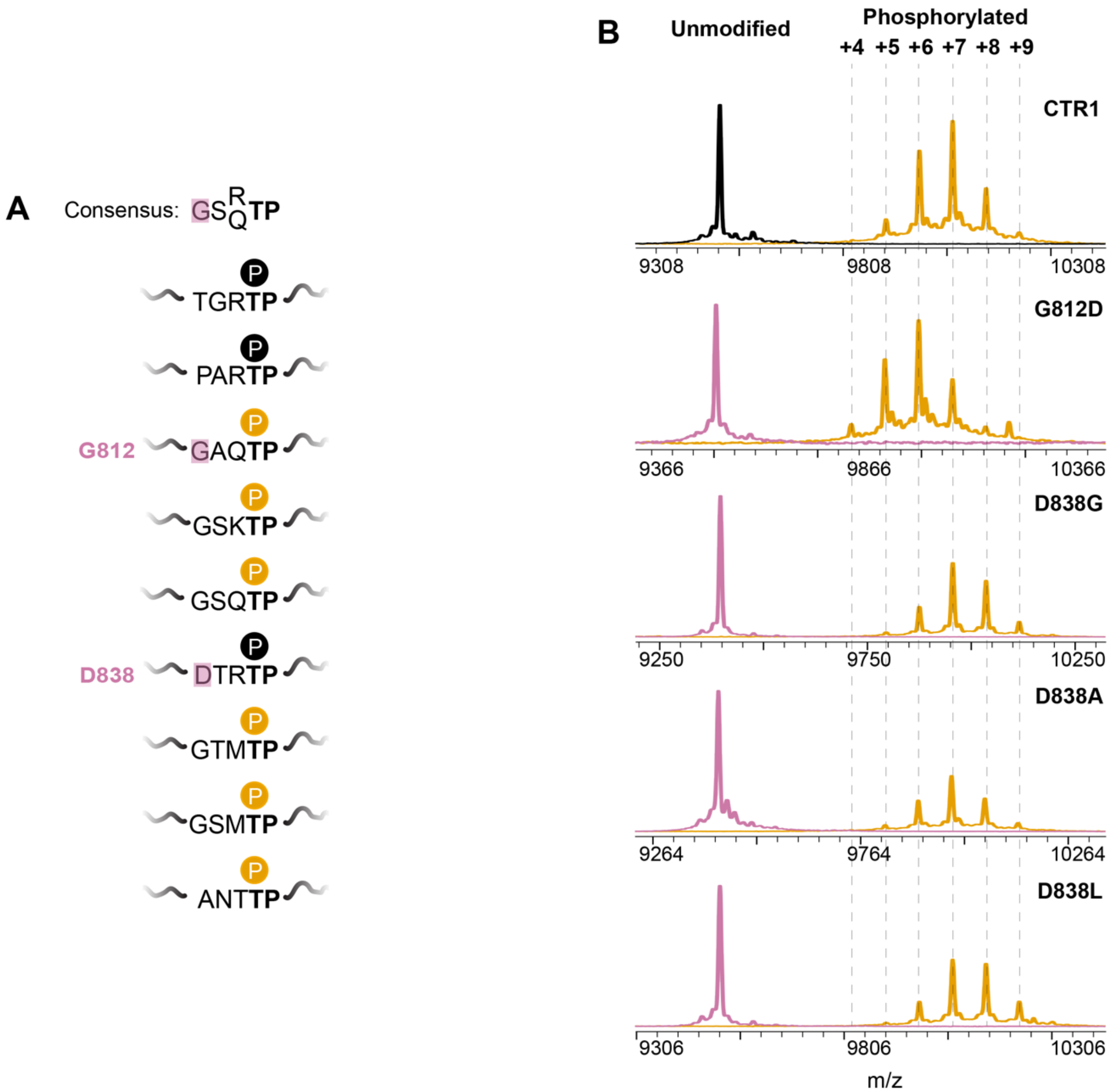

Figure S10: Sequence context influences the extent of CTR1 phosphorylation by P-TEFb (Gly/Asp substitutions). (A) Schematic representation of the SPT5 CTR1 region highlighting individual pentapeptide repeats containing Thr–Pro motifs. Residues targeted for mutagenesis are indicated. Phosphorylated threonine residues identified by NMR are marked with circled orange "P" symbols. (B) MALDI-TOF mass spectra of unmodified and phosphorylated wild-type and Gly/Asp-substitution mutant CTR1 constructs. In each spectrum, unmodified species are shown in black (wild type) or pink (Gly/Asp mutants), and phosphorylated species are shown in gold. Dashed lines and labels denote phosphorylation states and are included as visual guides to facilitate peak assignment.
